# *Plasmodium falciparum* GPCR-like receptor SR25 mediates extracellular K^+^ sensing coupled to Ca^2+^ signaling and stress survival

**DOI:** 10.1038/s41598-017-09959-8

**Published:** 2017-08-25

**Authors:** Miriam S. Moraes, Alexandre Budu, Maneesh K. Singh, Lucas Borges-Pereira, Julio Levano-Garcia, Chiara Currà, Leonardo Picci, Tomasino Pace, Marta Ponzi, Tullio Pozzan, Célia R. S. Garcia

**Affiliations:** 1 Departamento de Fisiologia, Instituto de Biociências, Universidade de São Paulo, Sao Paulo, SP 05508-090 Brazil; 2 Departamento de Parasitologia, Instituto de Ciências Biomédicas, Universidade de São Paulo, Sao Paulo, 05508-000 Brazil; 3 Foundation for Research and Technology-Hellas, Institute of Molecular Biology and Biotechnology, N. Plastira 100, GR 700 13 Heraklion, Greece; 4 Istituto Superiore di Sanita, Dipartimento di Malattie Infettive, Parassitarie ed Immunomediate, 0161 Roma, Italy; 5 Department of Biomedical Sciences, University of Padova, Institute of Neuroscience, Padova, Unit, National Research Council, Venetian Institute of Molecular Medicine, Padova, Italy

## Abstract

The malaria parasite *Plasmodium falciparum* is exposed, during its development, to major changes of ionic composition in its surrounding medium. We demonstrate that the *P*. *falciparum* serpentine-like receptor *PfSR25* is a monovalent cation sensor capable of modulating Ca^2+^ signaling in the parasites. Changing from high (140 mM) to low (5.4 mM) KCl concentration triggers [Ca^2+^]_cyt_ increase in isolated parasites and this Ca^2+^ rise is blocked either by phospholipase C (PLC) inhibition or by depleting the parasite’s internal Ca^2+^ pools. This response persists even in the absence of free extracellular Ca^2+^ and cannot be elicited by addition of Na^+^, Mg^2+^ or Ca^2+^. However, when the *PfSR25* gene was deleted, no effect on [Ca^2+^]_cyt_ was observed in response to changing KCl concentration in the knocked out (*PfSR25* ^−^) parasite. Finally, we also demonstrate that: i) *PfSR25* plays a role in parasite volume regulation, as hyperosmotic stress induces a significant decrease in parasite volume in wild type (wt), but not in *PfSR25* ^−^ parasites; ii) parasites lacking *PfSR25* show decreased parasitemia and metacaspase gene expression on exposure to the nitric oxide donor sodium nitroprusside (SNP) and iii), compared to *PfSR25* ^−^ parasites, wt parasites showed a better survival in albumax-deprived condition.

## Introduction

Malaria is responsible for 2–3 million deaths annually^1, 2^. The etiological agent of the most aggressive form of the disease, *Plasmodium falciparum*, upon RBC invasion resides inside a vacuole and undergoes various developmental changes, through the stages R (Ring), T (Trophozoite) and S (Schizont). Subsequent to nuclear division, several merozoites are generated, which upon release into the blood invade new RBCs^3^.

In the course of infection, *P*. *falciparum* is exposed to different microenvironments, in particular the ionic composition. We have shown previously that the *Plasmodium* vacuolar membrane is endowed with the capacity to generate a microenvironment around the parasites^4^ with a sufficient Ca^2+^ concentration to allow Ca^2+^ signaling to persist in the intracellular stage^5^.

In addition to the changes in Ca^2+^ levels, during the period spent in the mammalian host, *Plasmodia* must also deal with dramatic changes in K^+^ concentration, i.e. from 5 mM usually found in the blood stream, to 140 mM when the parasite resides inside the cytosol of host cells. Evidence has been presented over the last few years indicating that the changes in the different microambient of K^+^ concentration may affect several of the parasite functions. For example, exposure of liver-cell invading *P*. *berghei* parasites to high K^+^ stimulates cell invasion by the rodent malaria sporozoite^6^; along the same line, reducing K^+^ from 140 mM to 5 mM K^+^ in *P*. *falciparum* merozoites causes a rise in [Ca^2+^]_cyt_, leading to microneme secretion and merozoite egress from RBCs^7^. The microneme is a parasite organelle important for the parasite invasion and the release of its content was suggested to depend on [Ca^2+^]_cyt_ rise through PLC activation, but the molecular mechanisms that trigger K^+^ dependent effects in *Plasmodia* remain largely undefined. Here, we demonstrate that *PfSR25* (PlasmoDB number PF3D7_0713400), a member of a family of putative G protein-coupled receptors (GPCRs), which includes *PfSR1*, *PfSR10*, *PfSR12*, *and PfSR25* that were recently identified in *Plasmodium* database^8^, couples changes in extra parasitic K^+^ levels to PLC activation and intracellular Ca^2+^ mobilization. To the best of our knowledge, this is the only GPCR in any eukaryotic cell type with the capacity to respond to changes in environmental K^+^ concentration. Other possible functional roles of *PfSR25* have been investigated by comparing wild type (wt) and *PfSR25* ^−^ parasites. In particular, focusing on programmed cell death (PCD). It has been shown that many of the key apoptotic markers present in mammals do also exist in *Plasmodium* ^9^ and several research groups have demonstrated the participation of metacaspase in PCD of *Plasmodium* ^10, 11^. We hereby demonstrate that *PfSR25* ^−^ parasites are more susceptible to stress induced by NO production compared to wt cells, with a much larger increase in metacaspase expression. Finally, *PfSR25* ^−^ parasites also appear to be more sensitive than wt cells to chloroquine induced death and to stress induced by albumax removal from the medium. We also observed that *PfSR25* ^−^ parasites show higher inhibition in hemozoin formation after piperaquine treatment in comparison to wt parasites.

## Results

### *PfSR25* expression through RBC cycle

**Figure 1:**
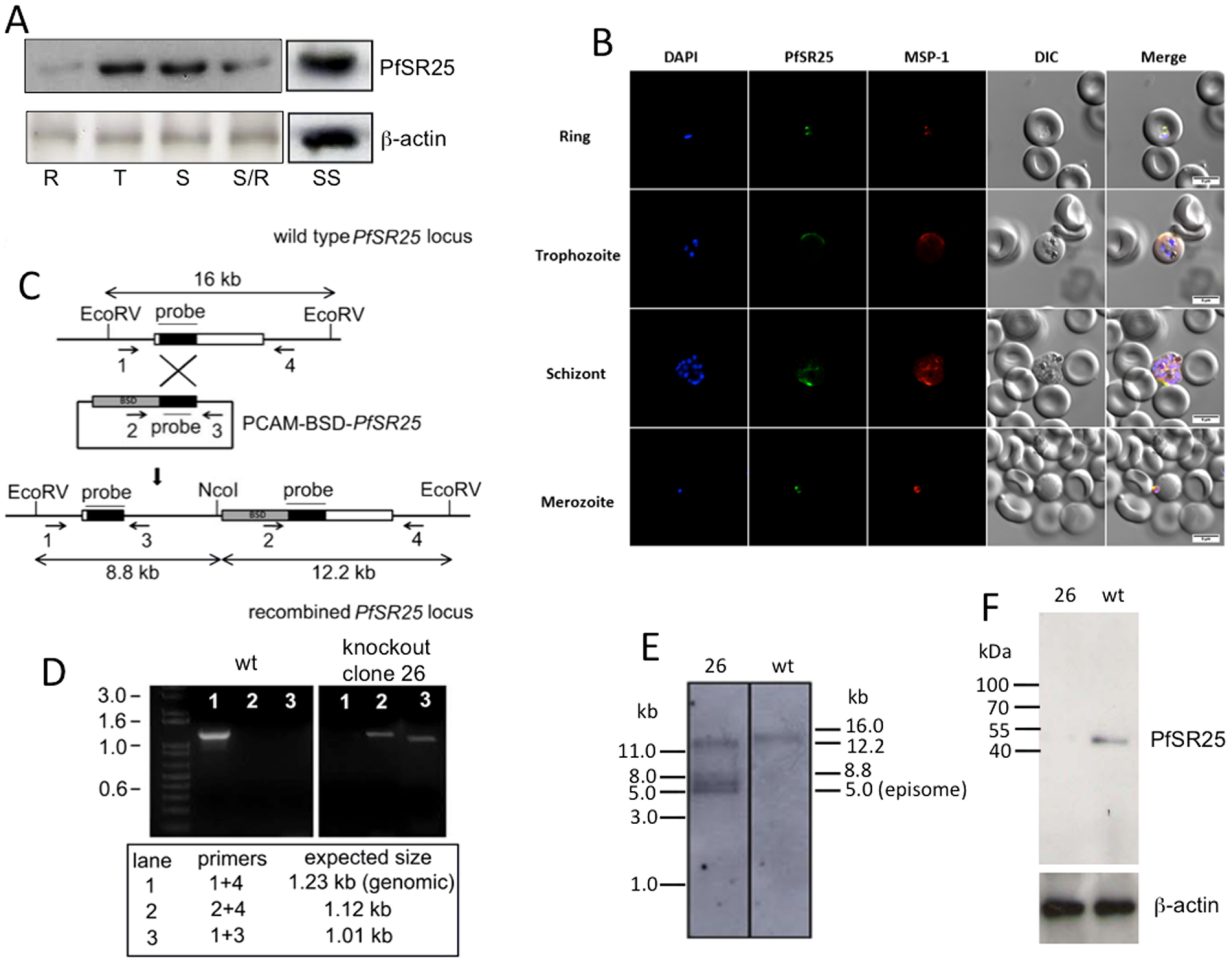
Intraerythrocytic expression of *PfSR25* and gene disruption. (**A)** Protein expression by WB. R: ring, T: trophozoite, S: schizont, SS: segmented schizont. Full length blots are indicated in the Supplementary Fig. S1B. (**B)** IFA of *PfSR25* during erythrocyte stages was done at ring; trophozoite; schizont and merozoite stage. PfMSP1 was used to confirm the co-localization of SR25. **(C)** Strategy for disruption of the *PfSR25 locus*. Primers used for the detection of integration events are indicated by numbered arrows (see materials and methods for the primer sequences), restriction sites by vertical lines. BSD, blasticidin resistance cassette; **(D)** PCR analysis of the *PfSR25* locus. The following primer pairs were used: primers 1 and 4 to detect wt locus (lane 1); primers 2 and 4 to detect integration at the 3′end of the insert (lane 2); and primers 1 and 3 to detect integration at the 5′end of the insert (lane 3). The expected sizes of the amplicons are indicated below the figure. **(E)** Southern blot analysis of the *PfSR25* locus in 3D7 wt parasites and *PfSR25* ^−^ (clone 26) parasites; wt locus 16 kb, integration of pCAM-BSD-*SR25* 12.2 kb and 8.0 kb, pCAM-BSD-SR25 episome 5.0 kb. **(F)** WB analysis of *PfSR25* protein from wt and clone 26 knockout (26) parasite with rabbit anti *PfSR25* antibody.

We have recently identified four potential GPCRs in *Plasmodium falciparum* genome and antibodies against these proteins have been generated. In particular, Western blots analysis with a specific anti-PfSR25 polyclonal antibody revealed the expression in the parasite blood stages (R, T and S) of a specific ~43 kDa band (consistent with the predicted molecular mass of *PfSR25*). Of interest, this band was stronger in more mature parasites, i.e., at the T and S stages and very faint at the R stage (Fig. 1A). Analysis of immunofluorescence images at different time points revealed the presence of *PfSR25* in R and T phase parasites with a diffused staining pattern. Interestingly, a particularly intense labeling was detected in schizonts, i.e. the stage prior to parasite egress from RBC. Finally, and most importantly, *PfSR25* was clearly detected in RBC-infective parasite stage, i.e. the merozoite. Co-localization with *Plasmodium* surface protein MSP-1 reveals that SR25 expression at parasite membrane (Fig. 1B).

To further investigate the functional role of *PfSR25* in *P*. *falciparum*, we generated and characterized knockout parasite clones (Fig. 1C–F). Gene disruption was verified by PCR (Fig. 1D) and Southern blot analysis (Fig. 1E) and protein expression by Western blot (Fig. 1F). As expected, neither the *SR25* gene nor the protein was detected in the *PfSR25* ^−^ samples.

In order to study the functional role of SR25 *in vivo* and not *in vitro* only, we also generated *PbSR25* knockout parasites in the rodent malaria model *P*. *berghei* (see Supplementary Fig. S1). Interestingly, in *P*. *berghei* transfected parasites that received the plasmid – thus GFP expressing (about the 0.3% of total parasites obtained after pyrimethamine selection) could be selected by fluorescence sorting by FACS but following purification and new injection in mice, they were not able to grow (see Supplementary Table S1).

### Changes in medium of KCl concentration modulate *P*. *falciparum* Ca^2+^ signaling

**Figure 2:**
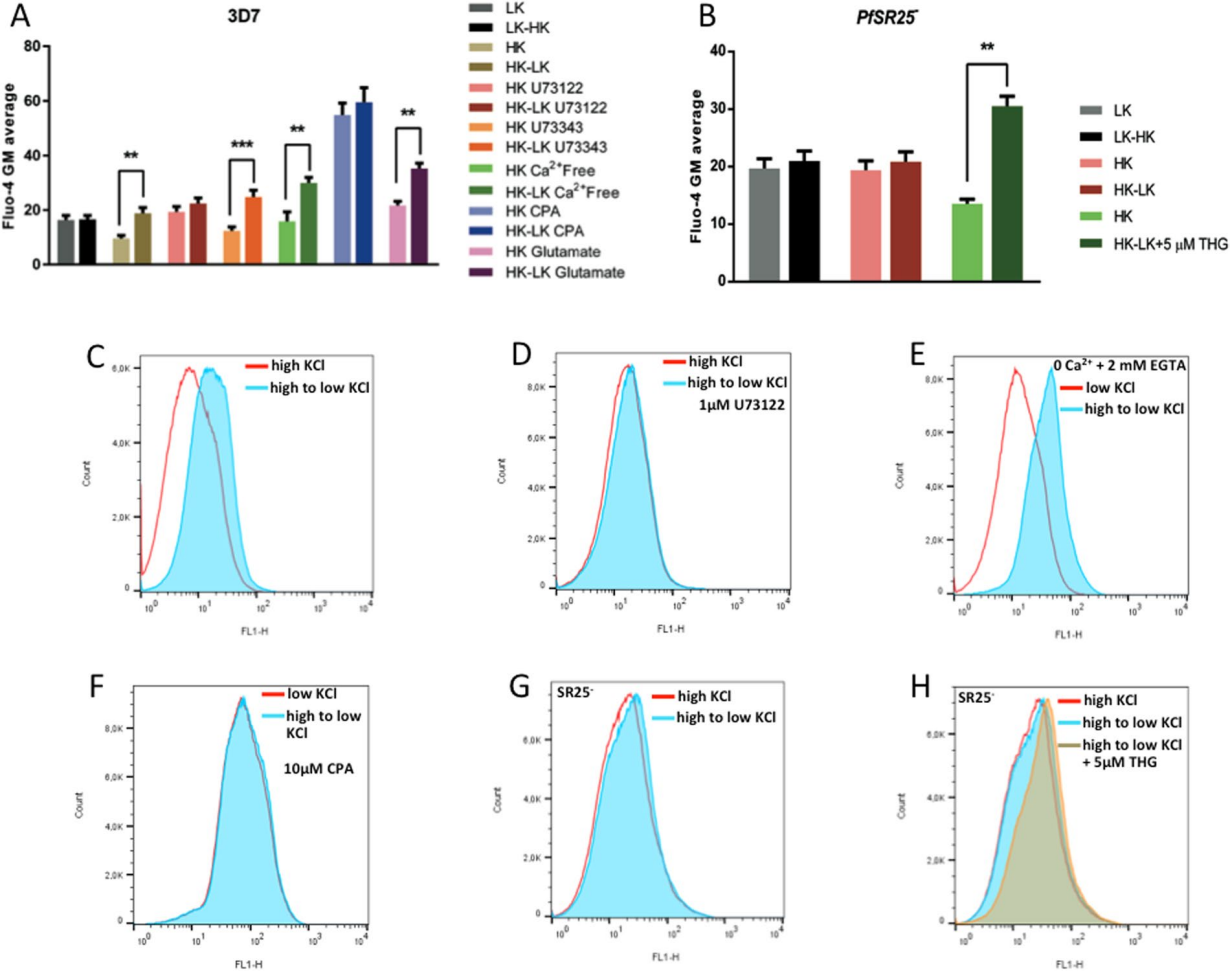
KCl and *PfSR25* are involved in [Ca^2+^]_cyt_ rise in isolated late trophozoites. Graphics of cytosolic calcium rise in terms of fluorescent shift in isolated wt and *PfSR25* ^−^ *P*. *falciparum* late trophozoites–early schizonts loaded with Fluo4-AM. **(A)** Cytosolic Ca^2+^ level detected by flow cytometry in Fluo-4 AM loaded wild type 3D7 parasite when exposed to high and then changed from high to low KCl environment. **(B)** Cytosolic Ca^2+^ level detected by flow cytometry in Fluo-4 AM loaded *PfSR25* ^−^ parasite when exposed to high and then changed from high to low KCl environment. Bar graphic of geometric means are plotted for each experiment and average Fluo-4 AM geometric means ± SEM of three independent experiments are shown. **p < 0.01, ***p < 0.001. **(C)** Overlay image of Fluo-4 AM shift when in high KCl and shift from high to low KCl; parasites were pre-incubated for 1 min with 1 µM U73122 **(D)** in high KCl buffer and measure the shift in both high and high to low KCl in the presence of 2 mM extracellular calcium. **(E)** A similar shift was observed in absence of extracellular Ca^2+^ when parasites were moved from high to low KCl. **(F)** In presence of 10 µM CPA, the fluorescent shift was abolished. Similarly rise in [Ca^2+^]_cyt_ was abolished when *PfSR25* ^−^ parasite were incubated with Fluo4-AM in high KCl containing buffer and changed to low KCl buffer **(G)**, but addition of 5 μM THG, a SERCA inhibitor in high to low KCl was able to elicit Ca^2+^ mobilization in the knockout parasite **(H)**.

In order to functionally characterize the role of *PfSR25*, the differential response to a series of different stimuli of wt and *PfSR25* ^−^ cells were compared. In particular, we addressed our attention to the Ca^2+^ signaling of the isolated late trophozoit/early schizont stage parasites. In a preliminary screen, we verified that some basic features of the Ca^2+^ signaling characteristics of *P*. *falciparum* are not affected by *PfSR25* ^−^, i.e., the basal Ca^2+^ level and the response to melatonin were indistinguishable in the two parasite strains (see Supplementary Fig. S2A and B). We next tested the effect on [Ca^2+^]_cyt_ when changing the extracellular K^+^ concentration. To this end *P*. *falciparum* cells, at the late T stages, were isolated from RBCs, loaded with the fluorescent Ca^2+^ indicator Fluo4-AM and incubated in the respective buffers with high K^+^/low Na^+^ (140 mM KCl, 5.4 mM NaCl) and low K^+^/high Na^+^ (5.4 mM KCl, 140 mM NaCl) supplemented with 2 mM CaCl_2_. We observed a fluorescent shift when parasites were transferred from high K^+^ to low K^+^ buffer but no difference in shift was found in the opposite condition i.e. low K^+^ to high K^+^ for wt parasites (Fig. 2A,C and see Supplementary Fig. S5). We also found that the KCl dose-response curve of the Ca^2+^-response was bell shaped as it was maximal at close to 30 mM KCl and then subsequent decreased at higher concentration of KCl (see Supplementary Fig. S2G).

The same protocol was then employed in the *PfSR25* ^−^, these parasites failed to respond to changes in KCl concentration when switched from high to low K^+^ buffer (Fig. 2B,G and see Supplementary Fig. S6). When the *PfSR25* ^−^ parasites were transferred from high to low K^+^ and treated with 5 μM thapsigargin (THG), an increase in [Ca^2+^]_cyt_ was however observed, indicating that internal Ca^2+^ stores are normally filled (Fig. 2H). We next investigated whether the [Ca^2+^]_cyt_ rise upon change in KCl depends on Ca^2+^ influx from the extracellular medium or on release from intracellular Ca^2+^ stores, or both. To this end, the same experiment of Fig. 2C was performed without extracellular Ca^2+^ (and in the presence of 2 mM EGTA). The KCl-induced [Ca^2+^]_cyt_ rise persisted under these latter conditions, clearly indicating that changes in KCl concentration in the extracellular medium mobilize Ca^2+^ from intracellular pools (Fig. 2E and see Supplementary Fig. S7). To further confirm this conclusion, cells were treated with cyclopiazonic acid (CPA, 10 µM) to deplete internal stores^12^ and no difference was observed in high or high to low [KCl]. Treatment with CPA was sufficient to completely prevent the KCl-induced [Ca^2+^]_cyt_ rise (Fig. 2F and see Supplementary Fig. S8). Along the same line, the PLC inhibitor U73122 abrogated the [Ca^2+^]_cyt_ rise caused by KCl (Fig. 2D and see Supplementary Fig. S9). On the other hand, the inactive analogue U73343 did not modify the [Ca^2+^]_cyt_ response to KCl (Fig. 2A and see Supplementary Fig. S10).

The [Ca^2+^]_cyt_ rise did not depend on osmotic effects or on Cl^−^ concentration, as: i) it is not elicited by addition of an equivalent dose of Na-Gluconate (Fig. 2A and see Supplementary Fig. S11); ii) the effect was indistinguishable using KCl or K-gluconate (Fig. 2A and see Supplementary Fig. S12). Finally, the effect is specific for K^+^, as changes in concentration of either Ca^2+^ or Mg^2+^ had no effect on [Ca^2+^]_cyt_ up to 5 mM, (see Supplementary Fig. S2C,D). Moreover when parasites where incubated in 5 mM and then KCl was increased to 50 mM we still observed a rise in [Ca^2+^]_cyt_ but similarly increase in concentration of other monovalent cation Na^+^ had no effect on [Ca^2+^]_cyt_ rise (see Supplementary Fig. S2E,F).

### PfSR25 expression and parasite volume regulation

**Figure 3:**
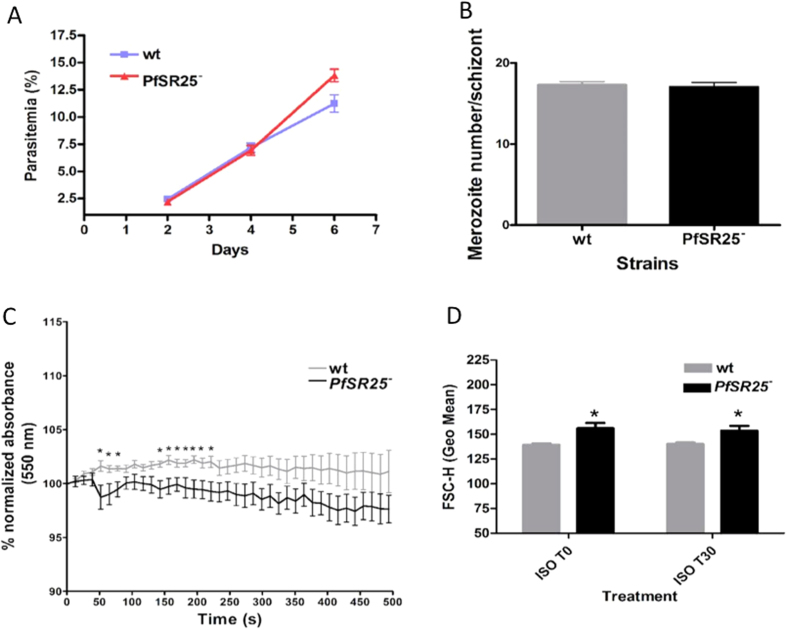
*PfSR25* is associated with *P*. *falciparum* volume changes. (**A)** Growth curve of *P*. *falciparum* wt and *PfSR25* ^−^ **(B)** Number of merozoites generated by *P*. *falciparum* wt or *PfSR25* ^−^ parasites. **(C)** Changes in volume of the isolated *P*. *falciparum* parasite (wt or *PfSR25* ^−^) upon exposure to hyperosmotic buffer, containing 50 mM D-sucrose **(D)** Changes in flow cytometric parameter of *P*. *falciparum* parasites (forward scattering, FSC-H, either *PfSR25* ^−^or wt strains, incubated in isosmotic buffer (ISO). The graph shows the mean ± S.E.M. of three independent experiments each performed in triplicate with the controls parasites (CTL). *p < 0.05 compared to the control. T0 - initial time; T30 - 30 minutes after treatment.

We then investigated whether the *PfSR25* ^−^ had other functional effects in the parasites. In particular, we found that: (i) the growth rate of *PfSR25* ^−^ parasites within RBC is indistinguishable from that of wt parasites (Fig. 3A); (ii) the merozoite number produced by schizonts is similar between the two parasite strains (Fig. 3B) and (iii) the invasion of RBC is not affected by *PfSR25* ^−^.

On the contrary, some differences were observed between wt and *PfSR25* ^−^ parasites in terms of volume regulation when exposed to hyperosmotic buffer. Figure 3C shows that volume decrease was clearly observed upon exposure to hyperosmotic medium in wt parasites while this were largely blocked in *PfSR25* ^−^ parasites. To further explore this result, forward light scatter (FSC) characteristics were used to quantify cell volume in isosmotic buffer as illustrated in Fig. 3D. Under these conditions, significant differences were observed in forward scatter between wt and *PfSR25* ^−^, the volume of mutant parasites being significantly larger than that of wt parasites.

### The *PfSR25* pathway modulates stress-induced parasite death

**Figure 4:**
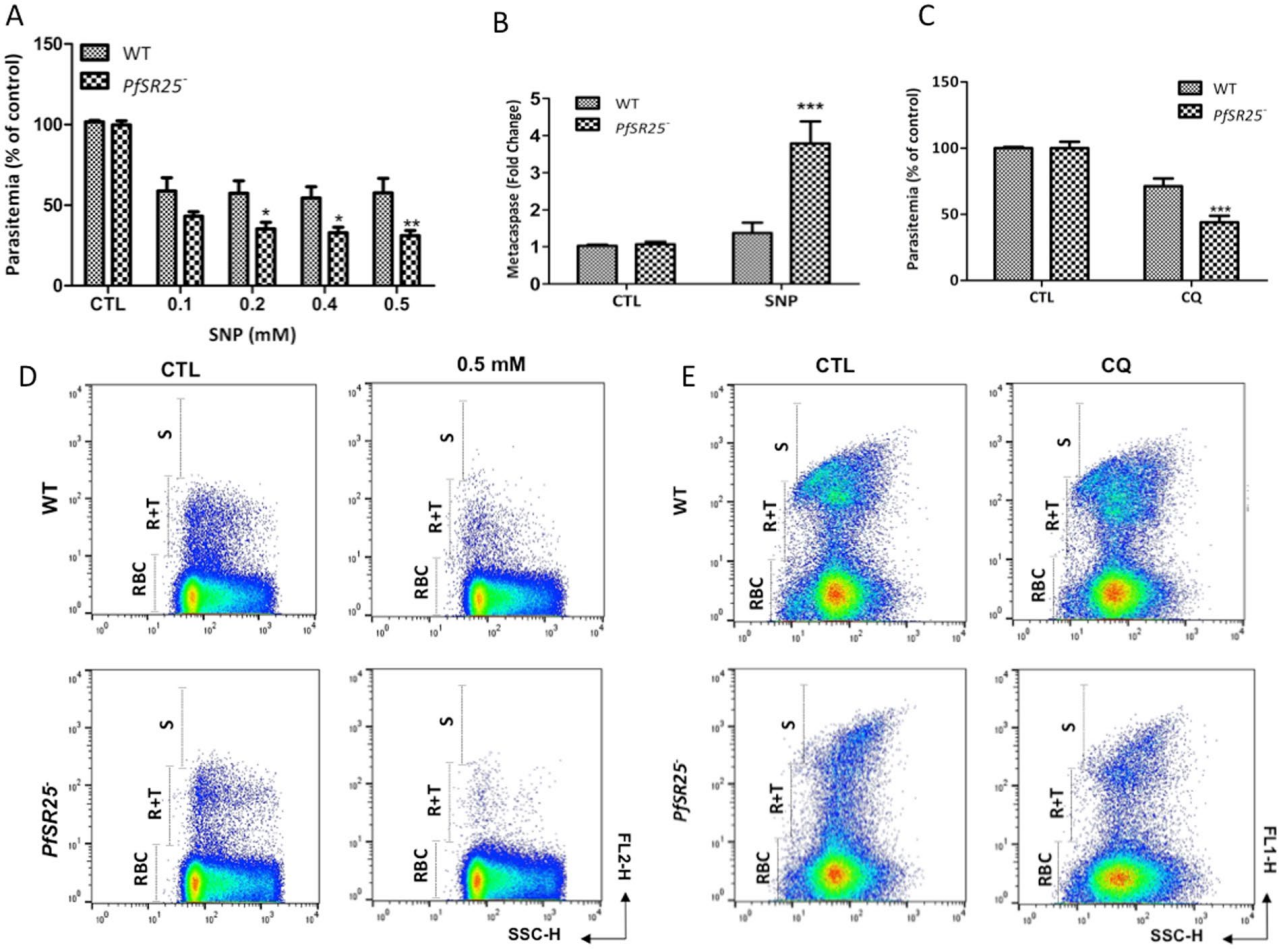
Modulatory effect of *PfSR25* on *P*. *falciparum* parasitemia mediated by SNP. (**A)** *P*. *falciparum* wt and *PfSR25* ^−^ were incubated under basal conditions (RPMI only) or with SNP at concentration of 0.1, 0.2, 0.4 and 0.5 mM for 24 hours. Parasite viability was evaluated using DHE. The data were expressed as percentages of those of the control conditions (100%), and represent the mean ± standard error of 3 different experiments performed in triplicate. (**B)** Analysis of the PfMCA1 by quantitative RT-PCR. RNA analysis was carried out 3 hours after SNP treatment. **(C)** Parasites were cultured in presence of 10 μM CQ for 24 h and parasitemia was determined by YOYO-1 staining. Representative FACS dot plot showing the fluorescence intensities for wt and *PfSR25* ^−^ parasites when treated with SNP **(D)** and CQ **(E)**. FL1 & FL2: fluorescence intensity; SSC: forward side scatter. The graph shows the mean ± S.E.M. of three independent experiments each performed in triplicate with the controls parasites (CTL) normalized to 100%. ***p < 0.001, *p < 0.05, **p < 0.01 compared to the control.

To determine the role of *PfSR25* in the resistance to toxic agents, *P*. *falciparum* synchronized 3D7 wt and *PfSR25* ^−^ parasites were treated with the NO donor, sodium nitroprusside (SNP) and the parasitemia were determined by flow cytometry. Exposure of *P*. *falciparum* to increasing doses of SNP (0–0.50 mM) demonstrated that *PfSR25* ^−^ is more sensitive to SNP toxicity than wt parasites (Fig. 4A and D). Giemsa stained smears showing the decreased parasitemia when treated with SNP (see Supplementary Fig. 3A and B). It has been shown that chloroquine is able to stimulate NO synthesis in human monocytes as well as in endothelial cells^13^. We thus next examined whether the survival effect was differentially affected by chloroquine treatment. The parasitemia observed in *PfSR25* ^−^ after exposure to chloroquine for 24 hr was consistently less than that produced by wt parasites (Fig. 4C and E) along with the giemsa stained smears (see Supplementary Fig. 3C), indicating decreased parasite viability. Reduced parasitemia could result from decreased cell proliferation and/or increased cell death. In addition, by analysing the levels of metacaspase after treatment of parasites for 3 hours with 0.5 mM of SNP, we found that *PfSR25* ^−^ parasites showed a 72% increase in metacaspase expression compared with wt parasites (Fig. 4B). To demonstrate that the action of SNP on *P*. *falciparum* was really due to its released NO, rather than other metabolite products, we have observed the ability of other NO donor at similar concentration in inducing cell death in *P*. *falciparum*. The effects of nitrosothiol derivative (SNAP) – NO donor on *P*. *falciparum* wt and *PfSR25* ^−^ were shown in Supplementary Fig. S4A. Treatment of parasites with SNAP at micromolar concentrations, decreased the parasitemia by 80% and 59%, respectively, in KO parasites compared to wt parasites, confirming the data obtained using SNP. These findings suggest that *PfSR25* may influence parasite susceptibility to SNP toxicity.

Nicotinamide adenine dinucleotide phosphate (NADPH) is a critical product of Glucose-6-phosphate dehydrogenase (G6PD), and is of central importance to the regulation of antioxidant system. Many studies have shown that G6PD deficiency renders cells extremely sensitive to oxidative stress^14–16^. Therefore, we were interested in studying the impact of *PfSR25* ^−^ in G6PD activity. G6PD activities were measured in samples from wt and *PfSR25* ^−^. The basal level of G6PD activity in *PfSR25* ^−^ was comparable to wt parasites, indicating that *PfSR25* ^−^ parasites respond under basal conditions with the same degree of NADPH production as wt parasites (see Supplementary Fig. S4C).

**Figure 5:**
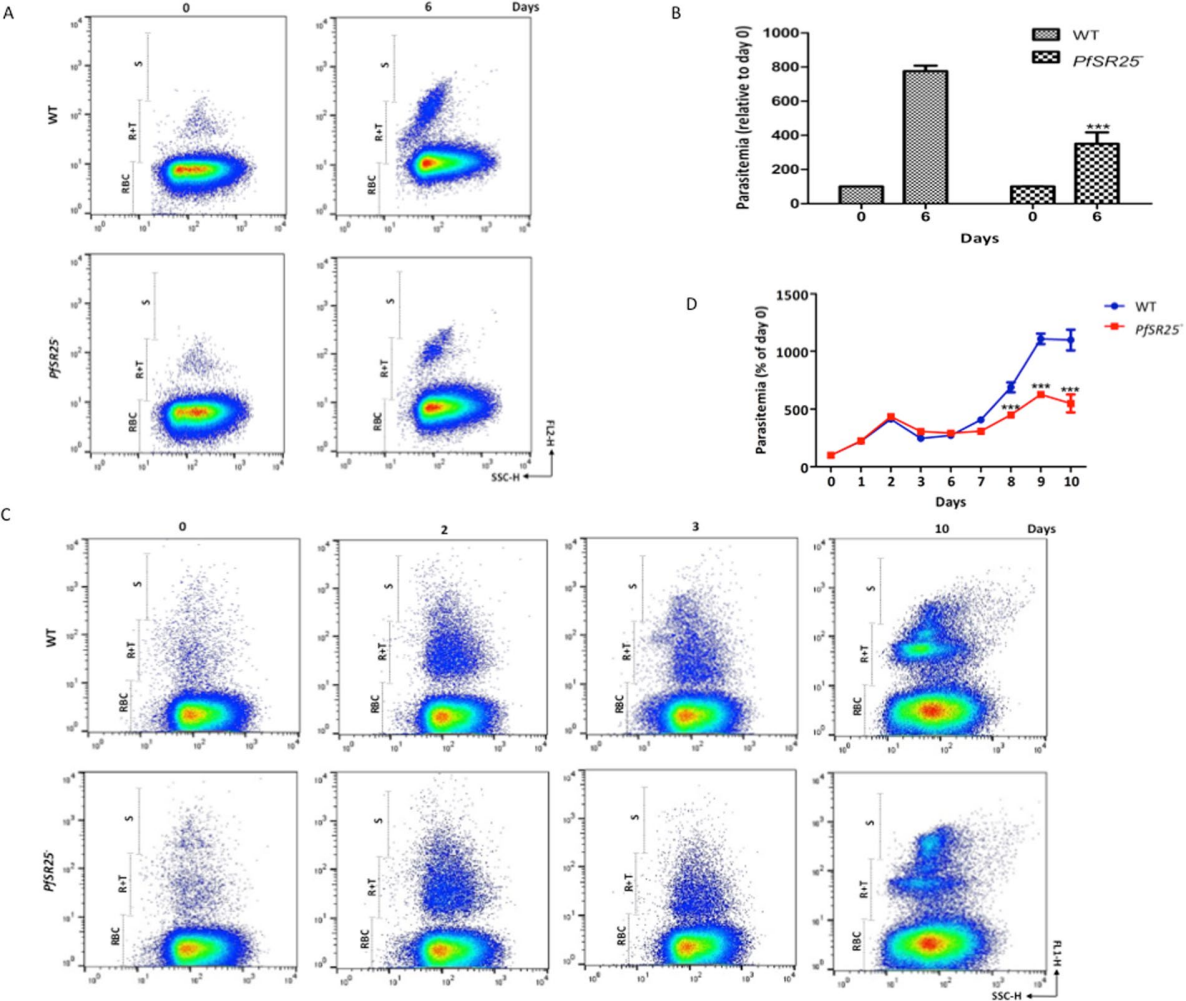
Wt parasites were more resistant to starvation than *PfSR25* ^−^ parasites. (**A**) Parasites were cultured in albumax-free media for 3 days. Albumax was reintroduced into albumax-free cultures on day 3. Parasitemia was determined by DHE staining after 0 and 6 days. (**B**) Histogram shows representative FACS dot plot showing the fluorescence intensities in Fig. 5A. (**C**) Parasites were cultured in albumax-free media for 3 days. Albumax was reintroduced into albumax-free cultures on day 3. Parasites were cultured for 10 d, with regular medium changed. Parasites were fixed in 2% PFA and the parasitemia was determined by YOYO-1 staining. Dot-plot was obtained after FACS analysis of YOYO-1 stained parasites. FL1 and FL2: fluorescence intensity; SSC: side scatter. (**D**) Graph showing the parasitemia during albumax-starvation experiment performed in Fig. 5C. The graphs show the mean ± S.E.M. of three independent experiments each performed in triplicate with the controls parasites (day 0) normalized to 100%. *p < 0.05, **p < 0.01, ***p < 0.001 compared with their respective day 0 parasites.

With the aim of better understanding how the *PfSR25* ^−^ parasites manage to survive under stress conditions we measured growth of PfSR25^−^ and wt parasites after albumax deprivation. For these experiments, *PfSR25* ^−^ and wt parasites were synchronized and maintained in albumax media for 24 h before switching to albumax-deprived media for 72 h without medium change. To assess whether all the remaining parasites were in fact dead, the percentages of parasites that continued to proliferate upon return to albumax-supplemented conditions was analysed. Parasites were switched back to albumax media for additonal 72 h. Medium was not renewed during the 72-hour incubation. We observed that parasitemia was significantly lower in *PfSR25* ^−^ parasites after being exposed to these stress conditions compared to wt cells (Fig. 5A,B and see Supplementary Fig. 4D). However, survival of parasites after albumax-deprivation was further ameliorated when the media was changed daily, even though the difference observed in parasitemia between *PfSR25* ^−^ and wt was still maintained (Fig. 5C,D).

**Figure 6:**
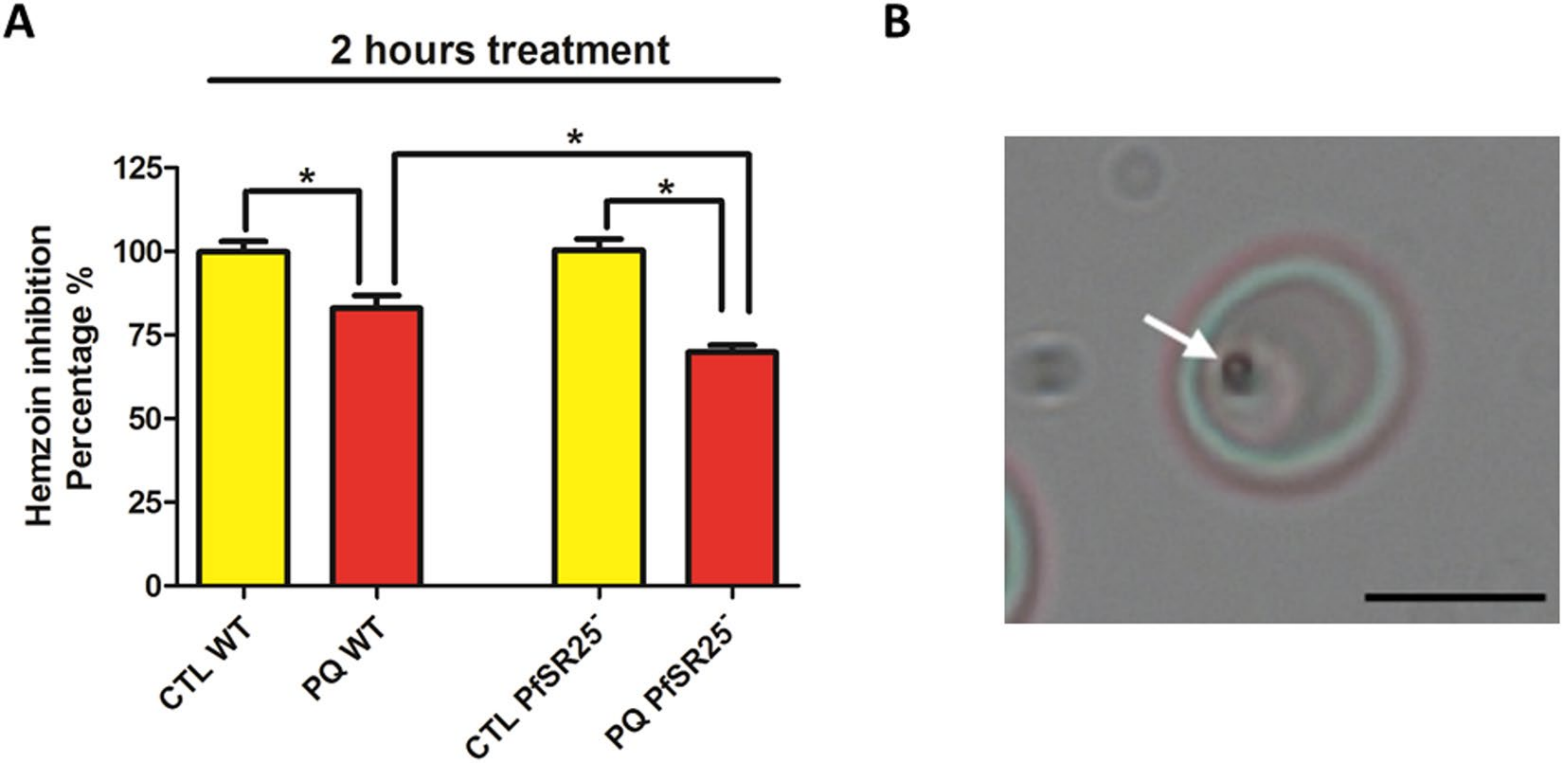
Percentage of inhibition of hemozoin crystal growth. (**A)** Both wt and *PfSR25* ^−^ trophozoite stage parasites were treated with 10 μM piperaquine for 2 hours and compared to control. (n = 3 ± SD) *P < 0.05. **(B)** Light microscope image of *P*. *falciparum* infected RBC; the objective used was a 100x N-Achroplan. The arrow indicates the hemozoin crystal with scale bar of 5 μm.

Direct role of SR25 when treated with antimalarial drug would give better perspective since its function is still not clear. We have treated both *PfSR25* ^−^ and wt trophozoites (32-34 hours post infection) with piperaquine (PQ) for 2 hours and measure the hemozoin formation. Interestingly, we found that *PfSR25* ^−^ parasites were very susceptible to drug treatment and after 2 hours, the hemozoin size formation was inhibited by approximately 69.9 ± 2.1% (Fig. 6).

## Discussion

Though separated by many million years of evolution, *Plasmodia* share with mammalian cells most of the functional characteristics of their signaling machinery. In particular, the malaria parasites use the same second messengers signaling principles of mammalian cells, i.e., the cAMP-protein kinase A and the IP_3_-Ca^2+^ signaling cascades^5, 17, 18^. Not surprisingly, however, many of the proteins involved in these processes are, at least in terms of primary sequence, totally different in the two types of cells. For example, though functionally and thanks to the work of several groups, conclusive evidence has been obtained in favor of the existence in Apicomplexa of an IP_3_ receptor linked to Ca^2+^ mobilization from stores^19–21^. Recently IP_3_R was identified in *T*. *brucei and T*. *cruzi* ^22, 23^ but no analogue of the mammalian protein has been found in the *Plasmodia* genome. Similarly, though searched intensively, proteins similar to mammalian GPCRs have been discovered in *Plasmodia* only very recently. We have described the expression in *Plasmodium falciparum* of four proteins that have the key characteristics of GPCRs, i.e., 7 potential transmembrane domains and relatively large extra and intracellular domains^8^. In the attempt of identifying the signaling pathway(s) controlled by these *Plasmodia* putative GPCRs (and their natural ligands) we discovered that one of them, *PfSR25*, is coupled to increases of [Ca^2+^]_cyt_ in response to changes of extracellular K^+^ concentrations. Changes in extracellular ion concentration (and in particular of K^+^) are relatively small (and either spatially very restricted, e.g. around active synapses, or linked to pathological conditions) in mammals, but are relatively frequent in unicellular eukaryotes and particularly in parasites such as *Plasmodia*, that spend part of their life cycle within cells (with high [K^+^]) and part in the blood stream, (with low [K^+^])^6^. By directly monitoring the [Ca^2+^]_cyt_ of isolated *Plasmodium falciparum* we here provide direct evidence for the existence of an extracellular K^+^ sensing mechanism. In particular we show that, among physiologically abundant cations, only changes in medium K^+^ concentrations are capable of modifying [Ca^2+^]_cyt_ in these cells. This finding is consistent with a previous report demonstrating that K^+^ was able to modulate, through undefined mechanisms, Ca^2+^ signaling in the blood stages of *Plasmodium falciparum* ^7^. Noteworthy, increases in extracellular K^+^ concentrations that are capable of rapidly increasing the parasite [Ca^2+^]_cyt_ are within the range that could be experienced by *Plasmodia* during their intra and extracellular life in the host. Under more physiological conditions, however, increases in [Ca^2+^]_cyt_ are observed also when *P*. *falciparum* are preincubated in medium with the physiological K^+^ concentrations of the blood (5 mM). One may thus speculate that *P*. *falciparum* undergo a rise in [Ca^2+^]_cyt_ both when invading the RBC (from low to high K^+^) and when released from RBC into the blood (from high to low K^+^). The effect of K^+^ is neither due to effects on membrane potential (and activation of voltage gated Ca^2+^ channels) nor to osmotic effects, as i) it is not elicited by equimolar concentration of other cations, ii) it occurs in the absence of extracellular Ca^2+^ and iii) it is inhibited by pre-depletion of intracellular Ca^2+^ pools and by addition of phospholipase C inhibitors, that block IP_3_ generation. Accordingly, we conclude that *Plasmodium* IP_3_ sensitive internal Ca^2+^ pools are the source of [Ca^2+^]_cyt_ rises induced by changes in K^+^ concentration. Most relevant, K^+^ dependent [Ca^2+^]_cyt_ increases are eliminated by knocking out the putative GPCR *PfSR25*. The simplest explanation for these data is that *PfSR25* is a K^+^ sensor itself, coupled via a G protein to phospholipase C activation. Alternatively, it may be speculated that, in order to act as a K^+^ sensor, *PfSR25* needs coupling to another, yet unidentified, *Plasmodium* specific protein.

One may have anticipated that an extracellular K^+^ sensor, never described before in any eukaryotic cell, should be of primary relevance in malaria parasite characteristics. On the contrary, and rather surprisingly, the key feature of *P*. *falciparum*, at least *in vitro*, is not significantly affected by genetic ablation of *PfSR25*. However, in the *in vivo* model *P*. *berghei*, the rodent malaria parasite, the deletion of the *PbSR25* orthologue gene was not possible. In fact, the percentage of KO parasites GFP expressing was very low (about 0.3% of the parasitemia) and purified mutant parasites (efficiently purified at 90%) injected to naïve mice did not lead to infection. However, if wt parasite contamination was at least the 20%, mutants could replicate with a multiplication rate of the 35% comparing to wild type parasites. The *PbSR25* ^−^ in *P*. *berghei* is not the first parasite line described in literature as non-vital. The database available at http://pberghei.eu/index.php also reports a similar result for the KO line of PBANKA_0915000 (AMA1), expressing as well GFP but impossible to clone (ID = 953) and purify (data

supported by Menard *et al*.)^24^. In particular, the invasion *in vitro* of RBC, parasite cell cycle progression and the release of merozoites is indistinguishable in wt and *PfSR25* ^−^ parasites. In *PfSR25* ^−^ cells we observed only marginal differences concerning volume regulation characteristics.

We thus considered two, non-mutually exclusive, explanations for this unexpected finding: i) KO of *PfSR25* may result in the activation of alternative pathways capable of compensating the absence of the K^+^ sensor; ii) the *in vitro* culture conditions may not mimic the *in vivo* situation where the K^+^ sensing mechanism may play a more essential role. To test this latter possibility we investigated whether *PfSR25* ^−^ parasites were more sensitive than wt cells under stress conditions. The parasites were thus challenged with NO (as induced by SNP treatment), the classical anti malaria drug chloroquine and albumax deprivation to induce parasite starvation. In all these three stress conditions *PfSR25* ^−^ parasites appear to be more sensitive than wt cells, suggesting that *PfSR25* could contribute to parasite survival under unfavourable conditions. Of interest, and particularly upon NO dependent stress, *PfSR25* ^−^ parasites responded to the toxic insult with a dramatic increase in metacaspase expression. Transcriptional profiling on lumefantrine (LM) selective resistant *P*. *falciparum* when kept in constant drug pressure for long time (16 months) has shown higher SR25 expression along with other transporters suggests that SR25 plays crucial role during drug-induced pressure (Mwai *et al*., 2012). Towards this end, we have measured the hemozoin size (Alves *et al*., 2015) in both wt and *PfSR25* ^−^ parasite after PQ treatment and our experiment shows that *PfSR25* ^−^ parasites were more sensitive to the PQ treatment than wt parasites and hemozoin formation was inhibited by 28% in *PfSR25* ^−^ parasites. This experiment is in agreement with Mwai *et al*. and gives direct evidence of SR25 during drug pressure.

**Figure 7:**
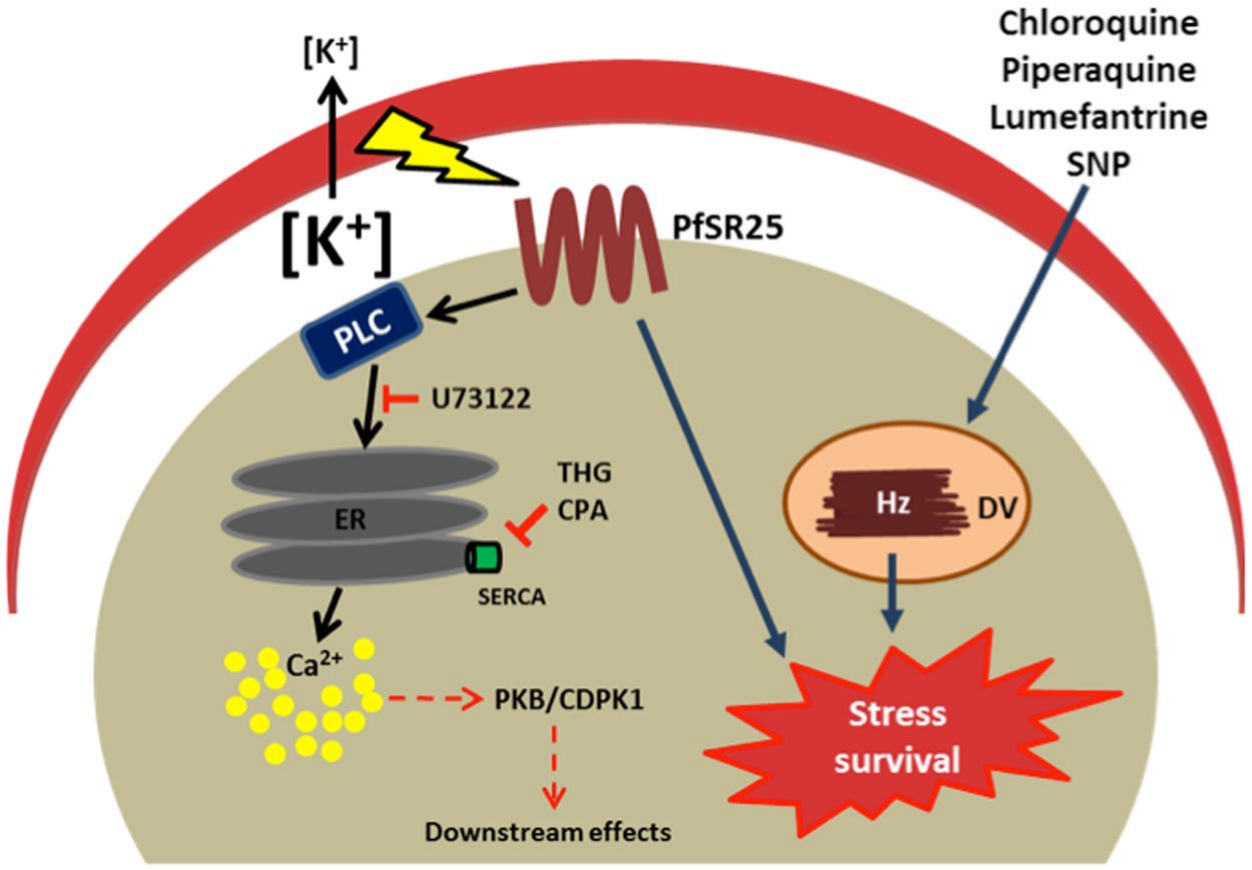
Schematic model of putative role of *PfSR25* in activation of Ca^2+^ signaling and stress survival: When *P*. *falciparum* parasite is exposed to low K^+^ environment, activation of *PfSR25* leads to PLC-mediated intracellular Ca^2+^ release from ER, which further activates downstream Ca^2+^-mediated signaling pathways such as PKB^25^ or CDPK1^26^. On the other hand, *PfSR25* ^−^ parasites display different sensitivity to the antimalarial drugs chloroquine, piperaquine and lumefantrine^43^ when compared to wt parasite. On top of that, recovery of wt parasite after SNP-mediated stress is higher in comparison to *PfSR25* ^−^. PLC - phospholipase C; ER – endoplasmic reticulum; Ca^2+^ - calcium ion; THG – thapsigargin; CPA – cyclopiazonic acid; SERCA – sarcoendoplasmic reticulum Ca^2+^ ATPases; PKB – protein kinase B; CDPK1 - calcium-dependent protein kinase 1; DV- digestive vacuole; Hz – hemozoin; SNP - sodium nitroprusside.

Based on our study, we have proposed the following model where we found that *PfSR25* is present on parasite surface. When the parasites are switched from high to low K^+^ environment, it elicits PLC-mediated Ca^2+^ release from parasite’s internal store. Cytosolic Ca^2+^ rise further activate multiple Ca^2+^-mediated intracellular signaling pathway necessary for new infection cycle^25, 26^. How *PfSR25* is transducing change in K^+^ to increase in Ca^2+^ could be a general mechanism for pathogen invasion in other biological models and deserves to be more fully exploited in the future. On the other hand, *PfSR25* ^−^ parasites are more susceptible to SNP treatment and exhibit reduced hemozoin formation when treated with antimalarial PQ (Fig. 7). The molecular mechanism(s) through which *PfSR25* improves parasite survival to stress is however not clear and requires further investigation. In addition, the involvement of two cellular mechanisms of action may explain the response of *PfSR25* ^−^ to starvation and chloroquine, since besides starvation, chloroquine also can activate autophagy process in *Plasmodium* ^27, 28^.

## Materials and Methods

### Materials

All cell culture reagents were obtained from Cultilab (Brazil). Fluo4-AM was from Molecular Probes. All other reagents were of the highest obtainable grade.

### Cell culture, synchronization and preparation of isolated parasites

The *P*. *falciparum* strain 3D7 was cultured at 37 °C in RPMI 1640 medium supplemented with 0.5% Albumax II (Gibco)^29, 30^. Cultures were grown under a 5% CO_2_, 5% O_2_, and 90% N_2_ atmosphere. The culture was synchronized with 5% sorbitol^31^ and parasitemia of 5–10% were obtained after 48 h of incubation. RBC-free parasites were prepared by using a previously published method^32^.

*Plasmodium berghei* ANKA 8417HP strain was used throughout the study^33^ and was maintained in CD1 mice (Harlan Sprague Dawley Inc). Purification of blood stages were performed as described^34^. Schizonts were isolated from cultures 23 h after set up and removal of uninfected red blood cells by density gradient centrifugation using Nycodenz.

### Protein extraction and western blot

For stage specific analysis of *PfSR25* expression, *in vitro* cultures were synchronized with two consecutive incubations in 5% sorbitol, and four protein points were collected: the rings were collected 6 h after synchronization, a mixture of trophozoites and early schizont forms were collected 24 h after synchronization, a mixture of late schizont forms and rings were collected 30 h after synchronization, and a mixture of late schizont forms and rings were collected 36 h after synchronization. At each time point, the cells were harvested, and the cell pellets were lysed for 60 s in ten volumes of 0.05% saponin prepared in PBS. The parasite pellet was recovered by centrifugation at 8,000 g for 10 min at 4 °C, washed and resuspended in lysis buffer (in mM): 50 Tris, pH 8.0, 150 NaCl, 5 EDTA, and 0.5% Nonidet P40 supplemented with the following mixture of protease inhibitors: 1 mM PMSF, 0.01 mM benzamidine, 10 µg ml^−1^aprotinin, 10 µg ml^−1^, leupeptin, 10 µg ml^−1^ pepstatin, 10 µg ml^−1^ chymostatin, 100 µM sodium orthovanadate, and 20 µM sodium fluoride. Particulate material was removed by centrifugation at 14,000 g for 30 min. Protein fractions were separated on 10% SDS-PAGE gels under reducing conditions, transferred to nitrocellulose, blocked with 5% powdered milk, and probed with PfSR25 specific polyclonal antibody (epitope sequence GTGEVKW) at a 1:2000 dilution. After extensive washing, the blots were incubated with HRP-conjugated goat anti-rabbit IgG (GE Healthcare) at a dilution of 1:10000 for 1 h, washed, and visualized using Plus ECL (GE Healthcare).

### Measurement of intracellular Ca^2+^ levels on *P*. *falciparum* synchronized culture

RBC at the late trophozoites-early schizont stage were centrifuged (2,000 rpm, 5 min), and the ~1 ml pellet was washed and isolated with 0.05% saponin in respective high (140 mM) or low K (5.4 mM) buffer so the parasites will remain in high or low potassium environment followed by loading with 10 µM Fluo4-AM for 30 min at 37 °C in high K^+^ (in mM:140 KCl, 5.4 NaCl, 0.8 MgSO_4_, 5.5 D-glucose, 50 MOPS, 2 CaCl_2_; pH 7.2); low K^+^ (in mM:5.4 KCl, 140 NaCl, 0.8 MgSO_4_, 5.5 D-glucose, 50 MOPS, 2 CaCl_2_; pH 7.2); K-gluconate (in mM:140 K-gluconate, 5.4 Na-gluconate, 0.8 MgSO_4_, 5.5 D-glucose, 50 MOPS, 2 CaCl_2_; pH 7.2) and Na-gluconate (in mM:5.4 K-gluconate, 140 Na-gluconate, 0.8 MgSO_4_, 5.5 D-glucose, 50 MOPS, 2 CaCl_2_; pH 7.2) respectively. Parasites were then washed three times in respective buffer and ~5 × 10^7^ parasites were transferred to high or low K^+^ containing vial prior to the experiment. For U73122 and U73343, parasites were pretreated for 1 min after Fluo4-AM loading. Fluo4-AM signal was measured for 2 min for each experiment by flow cytometry (FACSCalibur, Becton Dickinson, USA) excited with a 488 nm Argon laser and fluorescence emission was collected at 520–530 nm. All data were analyzed by FlowJo software with Histogram at y-axis and FL-1 at x-axis.

Cytosolic calcium dynamics were also monitored using Shimadzu spectrofluorophotometer (RF5301PC, Japan) in Fluo4-AM loaded parasite preincubated with 250 µM probenecid as described previously for calcium measurements^12^.

### Immunofluorescence confocal microscopy in *P*. *falciparum*

IFA was performed essentially as described previously^35^. For co-localization parasites were first incubated overnight at 4 °C with 1:100 rabbit anti-*PfSR25* in PBS containing 3% BSA and 0.01% Triton X-100. The secondary antibody anti-IgG rabbit Alexa 488 (Invitrogen) was used at 1:300 dilution in PBS containing 3% BSA for 1 h at room temperature. Parasites were rinsed in PBS, blocked in 3% BSA and 10% horse serum for 1 h and incubated with anti-MSP1 rabbit serum (1:500 dilution) for 1 h at room temperature followed by incubation with Alexa Fluor 546-conjugated anti-rabbit antibody (1:500 dilution) (Invitrogen). Parasites were incubated for 5 min with DAPI 1:1000 and washed three more times in PBS. Cells were mounted using Vectashield (Vector). Images were acquired with a Zeiss confocal microscope (LSM 780-NLO).

### Construction of knockout plasmid pCAM-BSD-KO-*PfSR25*

The parental plasmid pCAM-BSD was kindly donated by Christian Doerig^36^. A fragment from the *PfSR25* ORF (PlasmoDB PF3D7_0713400) was amplified by PCR and inserted between the *Pst*I and *Sac*I sites of the pCAM-BSD plasmid, which contains a cassette conferring resistance to blasticidin. A fragment spanning nucleotides 3 to 508 (numbered according to the PlasmoDB entry) was obtained using forward (ACTGCAGGGCTAAAAGGCACAAATTAAAG) and reverse (AGAGCTCCTAAATTCATGCATATCAAC) primers carrying *Pst*I and *Sac*I sites (underlined), respectively, and inserted into pCAM-BSD. DNA fragments were amplified from 3D7 genomic DNA by PCR, digested with the indicated restriction enzyme and cloned in pCAM-BSD.

The *PbSR25*(−) parasites were generated by introducing the drug-selectable cassette linked to a high expression GFP cassette in pBAT^37^ by double crossover. The *PbSR25* construct comprised a 809 bp PCR fragment of the 5′-FR of the gene amplified with primers PbSR25S1 and PbSR25S2, inserted in the restriction sites SacII-SpeI, and an 830 bp fragment of the 3′-FR amplified with primers PbSR25D1 and PbSR25D2 inserted in the sites ApaI-XhoI. The plasmid was digested with the enzyme PvuI before transfection. The gene was replaced via a double cross-over integrating the recyclable hDHFR-yFcu cassette which encodes anti-folate resistance. GFP expression was regulated by the PbHSP70 promoter. After transfection about 2000 GFP-fluorescent red blood cells were sorted and intravenously injected into two CD1 mice. When parasitaemia was established, only the 5% of total parasitemia was depending on GFP-positive parasites. Sorting and new infections were repeated two more times, but no parasitemia was detected. All primer sequences are available upon request.

The animal work has been authorized by the Italian Ministry of Health, according to the Legislative Decree 116/92 which implemented the European Directive 86/609/EEC on laboratory animal protection. Animals used in this study were haused and treated according to Legislative Decree 116/92 guidelines.

### Parasite transfection

Transfection was carried out by the electroporation of ring stage parasites at 5-10% parasitemia with 100 µg of plasmid DNA. Following transfection, parasites were maintained in drug-free medium for 48 h at which time positive selection was initiated by the addition of 2.5 µg/ml blasticidin to the medium. Blasticidin-resistant parasites appeared after 3 to 6 weeks of culture, and their genotype was evaluated by PCR to verify that integration had occurred at the target locus. The parasites were then cloned by limiting dilution in 96-well plates (0.5 to 1 parasite/well)^38^ and their genotype was analyzed using PCR.

### Genotype characterization by PCR

For the detection of integration of the plasmid pCAM-BSD-KO-*PfSR25* into genomic DNA the following primers were used: primer 1, TACATATATACATGCCTTGAAC; primer 2, GAACATATTTATTAAACTGCAG; primer 3, TCACTAAAGGGAACAAAAGCT; and primer 4, GAAAGGTAATTTGGTCTGTAC. Primers 1 and 4 correspond to *PfSR25* sequences, and primers 2 and 3 correspond to pCAM-BSD vector sequences flanking the insertion site.

### SNP experiments

Parasites from each lineage were incubated with SNP at a concentration of 0 (basal), 100, 500, 1000 or 2000 µM for 6 hours at 37 °C in a 5% CO_2_ humidified atmosphere; both the selected concentration(s) and the specific duration(s) of incubation are specified in the results section. For FACS experiments, cells were grown in a 24-well plate; whereas parasites were grown in 25 cm^2^ plastic flasks for DNA and RNA extraction.

### Total RNA isolation

Total RNA was extracted from various parasites cultures using TRIZOL (Invitrogen, USA). Synchronized parasites, with or without *in vitro* stimulation, were lyzed directly in a culture flask by adding TRIZOL. The homogenates were incubated for five minutes at room temperature to permit complete dissociation of nucleoprotein complexes. Next, 0.2 ml chloroform was added per millilitre of extraction reagent; the mixture was shaken vigorously for 15 seconds and incubated for two to three minutes at 4 °C. Centrifugation separated the homogenates into two phases: a lower brown phenol/chloroform phase and a colourless upper aqueous phase containing RNA. The aqueous upper phase was transferred to a fresh tube, and 0.5 ml isopropanol was added per 1 mL of TRIZOL. This mixture was incubated at room temperature for 10 minutes and then centrifuged for 10 minutes at 12,000 × *g*. Supernatants were removed, and the RNA pellet was washed once with 75% ethanol by vortexing and subsequent centrifugation at 7500 × g for five minutes at 4 °C. The extracted RNA in the pellet was air dried and dissolved in DEPC-H_2_O for use in qRT-PCR analysis.

### Quantitative Real-Time Polymerase Chain Reaction (qRT-PCR)

RNA was extracted from synchronized schizont 46–48 h post-invasion. Total RNA was isolated and purified according to manufacturer’s instructions using TRIZOL® reagent (Invitrogen) and measured using a NanoDrop 2000c spectrophotometer (Thermo Scientific). Equal amounts of total RNA were synthesized into cDNA using a First-Strand cDNA Synthesis kit (Invitrogen). ABgene Absolute qPCR SYBR Green Master Mix (ABgene, Surrey, UK) with ROX dye was used for all PCR protocols. The following primer pairs from Extend were used: Metacaspase (PF3D7_1354800): forward 5′-GCTGTTGTAGATAGTTGTAATTCTGG-3′, reverse 5′-GGTGCAATCTGTCCTGTGTTAAC-3′. serine tRNA-ligase (PF3D7_0717700): forward 5′-TGGAACAATGGTAGCTGCAC-3′, reverse 5′-TCATGTATGGGCGCAATTT-3′. qRT-PCR reactions were run in triplicate, in 96-well plates on an ABI Prism^®^ 7900HT real-time PCR machine. Thermal cycling conditions were as follows: one cycle at 50 °C for 2 min for enzyme activation; one cycle at 95 °C for 15 min for enzyme activation; forty cycles at 95 °C for 15 sec for Denaturation and 60 °C for 1 min for the Anneal/Extension step. Threshold cycle number (Ct) of gene was calculated, and serine tRNA-ligase was used as reference gene. Delta–delta Ct values of genes were presented as relative fold induction.

### Volume and parasitemia assessment via FACS

Flow cytometry analysis was performed essentially as described previously^39^. For volume assessment, cells were washed in PBS buffer (pH 7.4). Infected-erythrocytes were stained with dihydroethidium (DHE-Sigma) at a 1:1000 dilution. After 30 min, samples were diluted 1:5 and measured by flow cytometric analysis (FACSCalibur, Becton Dickinson, Heidelberg, Germany). The fluorescence intensity of dihydroethidine was measured in fluorescence channel FL-2. Cell volume was determined by forward-and side-scatter analysis of unstained cells. For parasitemia assessment, parasites were incubated at 0.1% parasitemia in 0.5 mL RPMI 1640 containing 0.5% albumax for 6 days. Infected RBCs were collected each 48 h, stained with YOYO-1 and parasitemia was assessed by FACS^40^. To assess the number of merozoites in each schizont, a synchronized culture was maintained in RPMI containing 0.5% albumax until the development in segmented schizonts, at least 20 schizonts were assessed in each slide. The experiment was repeated 3 independent times.

### Volume assessment via spectrophotometer

The protocol used was adapted from^41^. Parasites were synchronized with 5% sorbitol. 24 h after synchronization, parasites were isolated from RBCs with saponin 0.3% in PBS and incubated in Buffer A with 2 mM CaCl_2_ at 37 °C in Flexstation 3 fluorimeter (Molecular Devices). 50 mM sucrose in buffer A was added and absorbance at 550 nm was assessed upon sucrose addition. Data was normalized by the first absorbance reading of each graphic.

### Measurement of glucose 6-phosphate dehydrogenase (G6PD)-activity

The levels of G6PDH- activity were performed according to the manufacturer’s protocols for G6PD assay kit (ab102529, Abcam, Cambridge, MA, USA.]. Sorbitol synchronized trophozoite stage parasites (haematocrit: 2.5%, parasitaemia: 5%) under normal culture conditions were washed with PBS twice and 10^8^ RBC were seeded into a poly-L-lysine-coated 96 well plate. Samples and their controls, and the standard curve for NADPH were read at 450 nm on the FlexStation3.

### Hemozoin area measurements in *P*. *falciparum*-infected RBCs

The hemozoin size measurement was performed as previously described^42^. Briefly, *P*. *falciparum*-infected erythrocytes (wild type and *PfSR25* ^−^ parasites) were incubated with 10 µM piperaquine for 2 hours at 37 °C. After, the parasites were washed with PBS and placed in a slide that had been previously pre-treated with poly-L-lysine to ensure adhesion of the cells. The images were acquired in an Axio Scope A.1 microscope (Carl Zeiss) and analyzed with Zen software. The objective used was a 100x N-Achroplan with immersion oil.

### Statistical analyses

Each experiment was performed at least three times. The data shown are those of a representative experiment. Statistical significance was evaluated with One-Way ANOVA test and Newman-Keuls post-test, and all comparisons with p value less than 0.05 (p < 0.05) were considered statistically significant. The data are expressed as the mean ± SEM.

## Electronic supplementary material

Supplementary material
