# Supplementary material for: Plasmodium falciparum GPCR-like receptor SR25 mediates extracellular K+ sensing coupled to Ca2+ signaling and stress survival
Source: Sci Rep. 2017 Aug 25;7:9545. doi: 10.1038/s41598-017-09959-8 (PMC5573319; doi:10.1038/s41598-017-09959-8)
Supplement: Supplementary file 1 — Supplementary material [file 41598_2017_9959_MOESM1_ESM.pdf]

Supplementary Information

Plasmodium falciparum GPCR-like receptor SR25 mediates extracellular K^+ sensing coupled to Ca^{2+} signaling and stress survival

Miriam S. Moraes[1,2,*], Alexandre Budu[1,2,*], Maneesh K. Singh[1,2,*], Lucas Borges-Pereira[1,2], Julio Levano-Garcia[1], Chiara Currà[1,3], Leonardo Picci[4], Tomasino Pace[4], Marta Ponzi[4], Tullio Pozzan[5] and Célia R. S. Garcia[1]

[1]Departamento de Fisiologia, Instituto de Biociências, Universidade de São Paulo, São Paulo, SP, 05508-090, Brasil.

[2]Departamento de Parasitologia, Instituto de Ciências Biomédicas, Universidade de São Paulo, São Paulo, 05508-000, Brasil.

[3]Foundation for Research and Technology—Hellas, Institute of Molecular Biology and Biotechnology, N. Plastira 100, GR 700 13 Heraklion, Greece.

[4]Istituto Superiore di Sanita`, Dipartimento di Malattie Infettive, Parassitarie ed Immunomediate, 0161 Roma, Italy.

[5]Department of Biomedical Sciences, University of Padova, Institute of Neuroscience, Padova, Unit, National Research Council, Venetian Institute of Molecular Medicine, Padova, Italy.

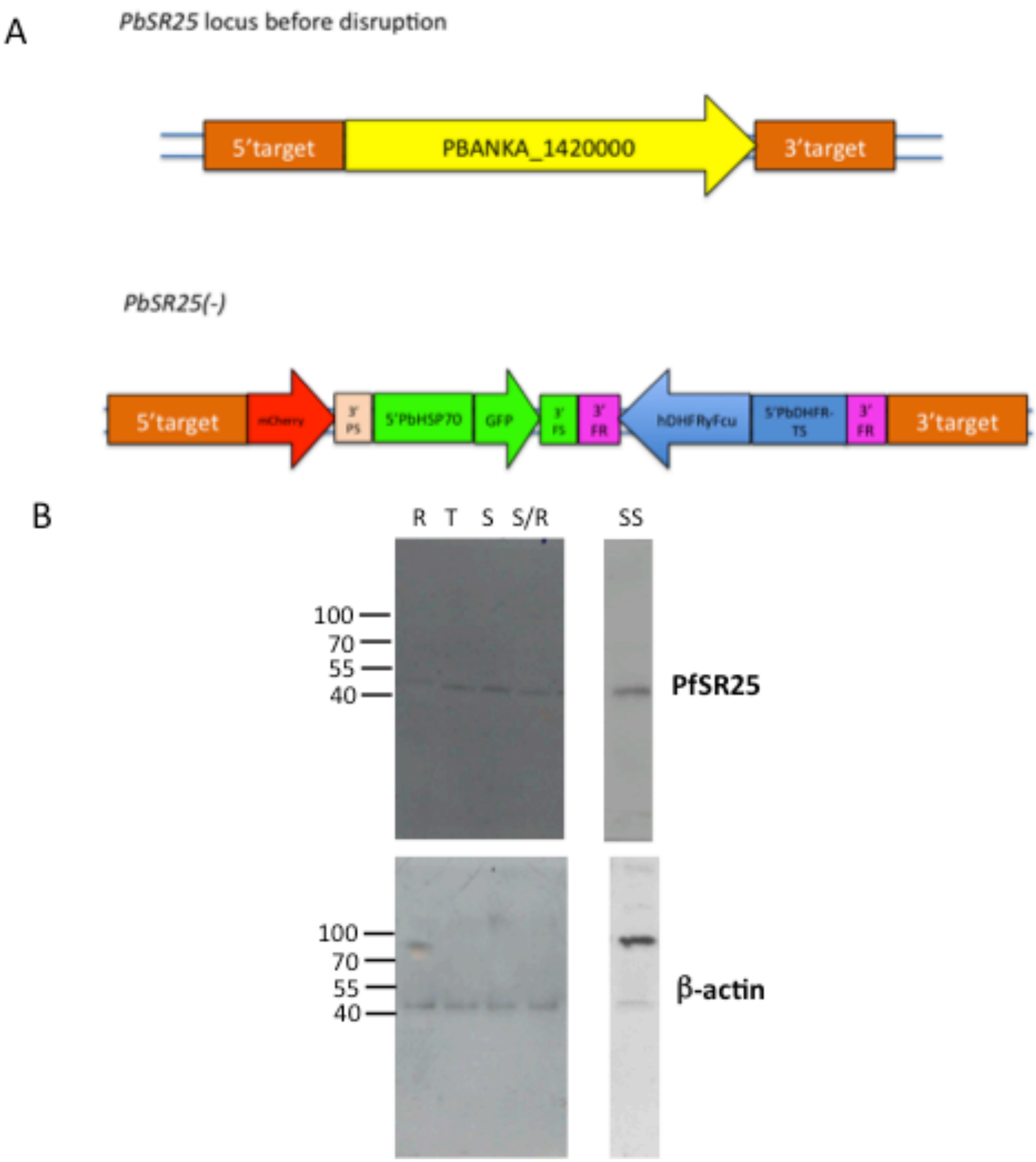

Fig. S1. (A) Schematic representation of the WT PBANKA_1420000 locus. 5' and 3' targeting fragments are indicated in orange. (ORF in yellow). Generation *PbSR25(-)* and the modified locus of *orp2(-)*. 5' and 3' targeting fragment (orange) are indicated for integration via double crossover of a fragment containing the drug selectable cassette *hDHFR* fused to yeast *Fcu* (blue) and the gene encoding GFP under the control of the HSP70 promoter (green). Additional sequences of the plasmid vector pBATSIL6 are indicated in red (mCherry) and pink (3' flanking region, FR). **(B)** Full length blot of *PfSR25* from R, T, S and SS depicted in **Fig. 1A**. Anti-actin control for segmented schizont is also staining a stronger 70 kDa band (probably due to actin ubiquitination) in parasite was preferentially used as a loading control.

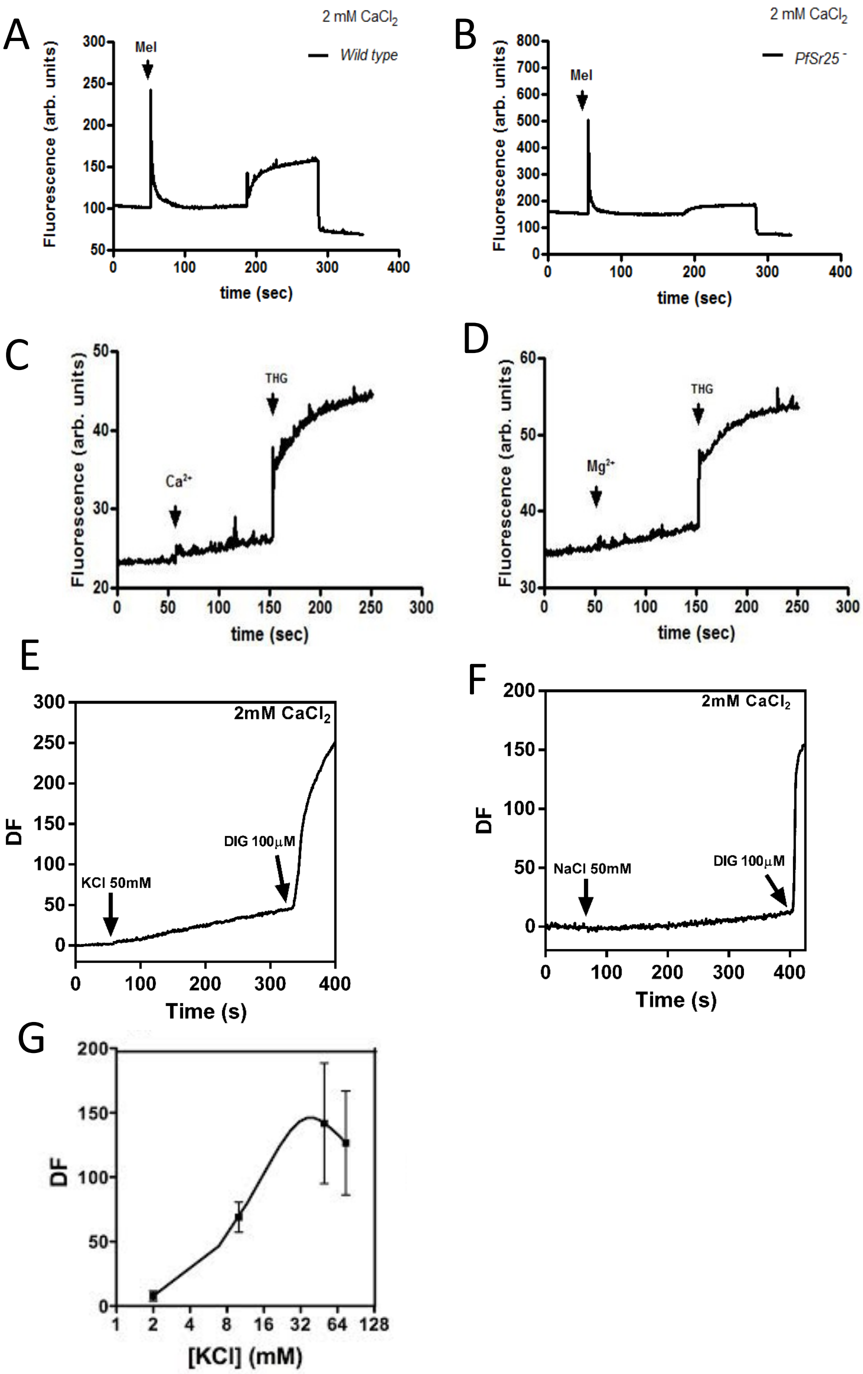

Fig. S2. **Calcium response in *P. falciparum* trophozoite.** Kinetics of cytosolic calcium signals $[Ca^{2+}]_i$ induced by 100 nM melatonin in *P. falciparum* wt **(A)** and *PfSR25*$^-$ parasites **(B)**. Ca^{2+} 5 mM **(C)** and Mg^{2+} 5 mM **(D)** addition to isolated *P. falciparum* parasites in buffer in nominally free Ca^{2+} buffer do not elicit a strong $[Ca^{2+}]_{cyt}$ rise. Thapsigargin (THG) was added at 5 μM, as a positive control. Addition of 50 mM KCl leads to a calcium rise **(E)**, but 50 mM NaCl **(F)** was unable to elicit any intracellular calcium rise. **(G)** The dose-response curve of the Ca^{2+} with KCl is bell shaped as it was maximal at around 30 mM KCl and then decreased at higher concentrations.

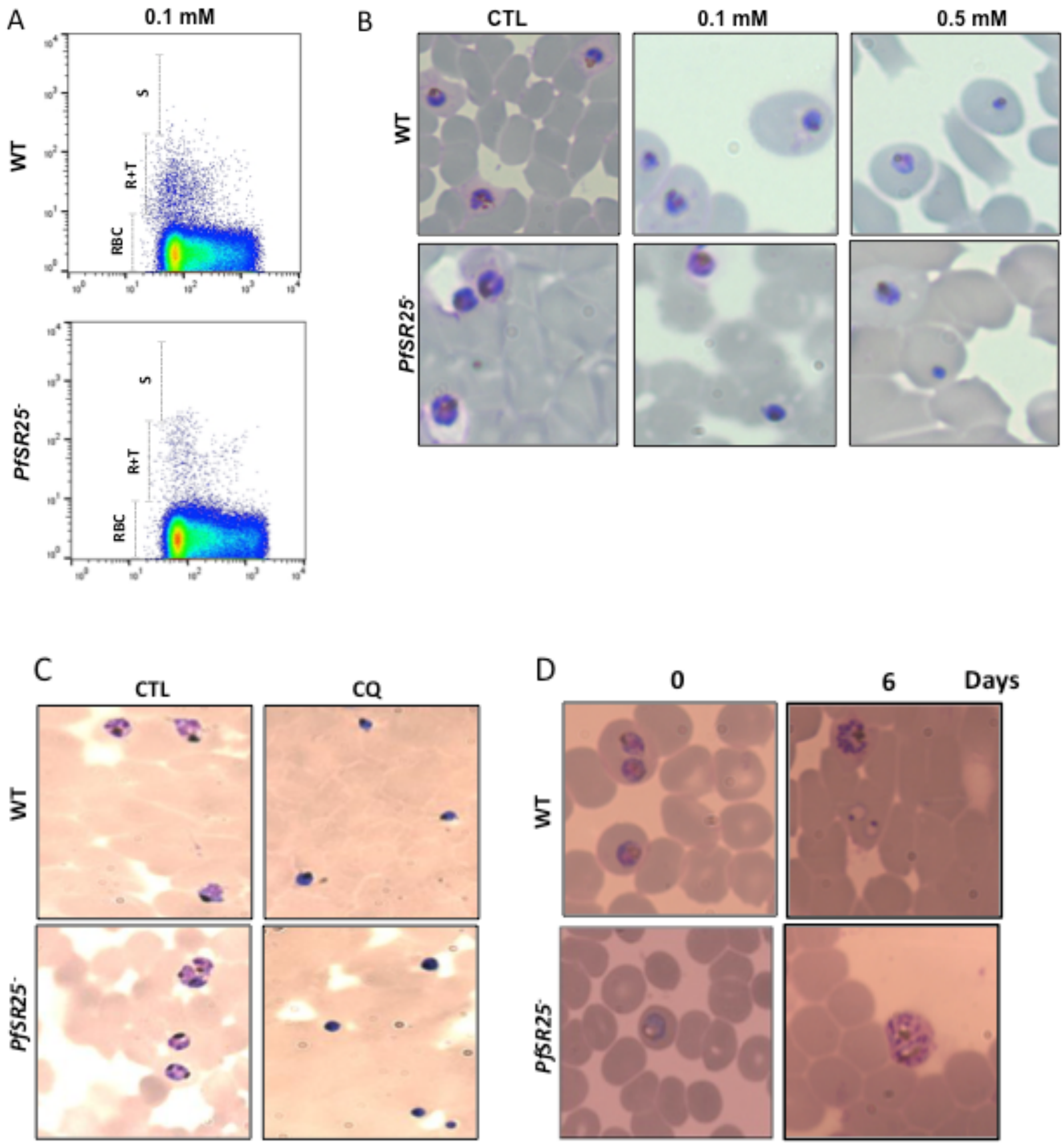

Fig. S3. SNP mediated parasitemia and starvation effect on wt and *PfSR25*⁻ parasite. (A) Dot-plot obtained after FACS analysis of DHE-stained for 0.1 mM treatment. FL2: fluorescence intensity; SSC: side scatter. **(B)** Giemsa-stained thin-blood smears were prepared for 0.1 and 0.5 mM SNP after 24 h treatment. **(C)** Parasites were cultured in 10 µM CQ for 24 h and digital image *of* Giemsa-stained thin blood smears. **(D)** Parasites were cultured in albumax-free media for 3 days. Albumax was reintroduced into albumax-free cultures on day 3. Giemsa-stained thin blood smears were prepared for 0 and 6 days.

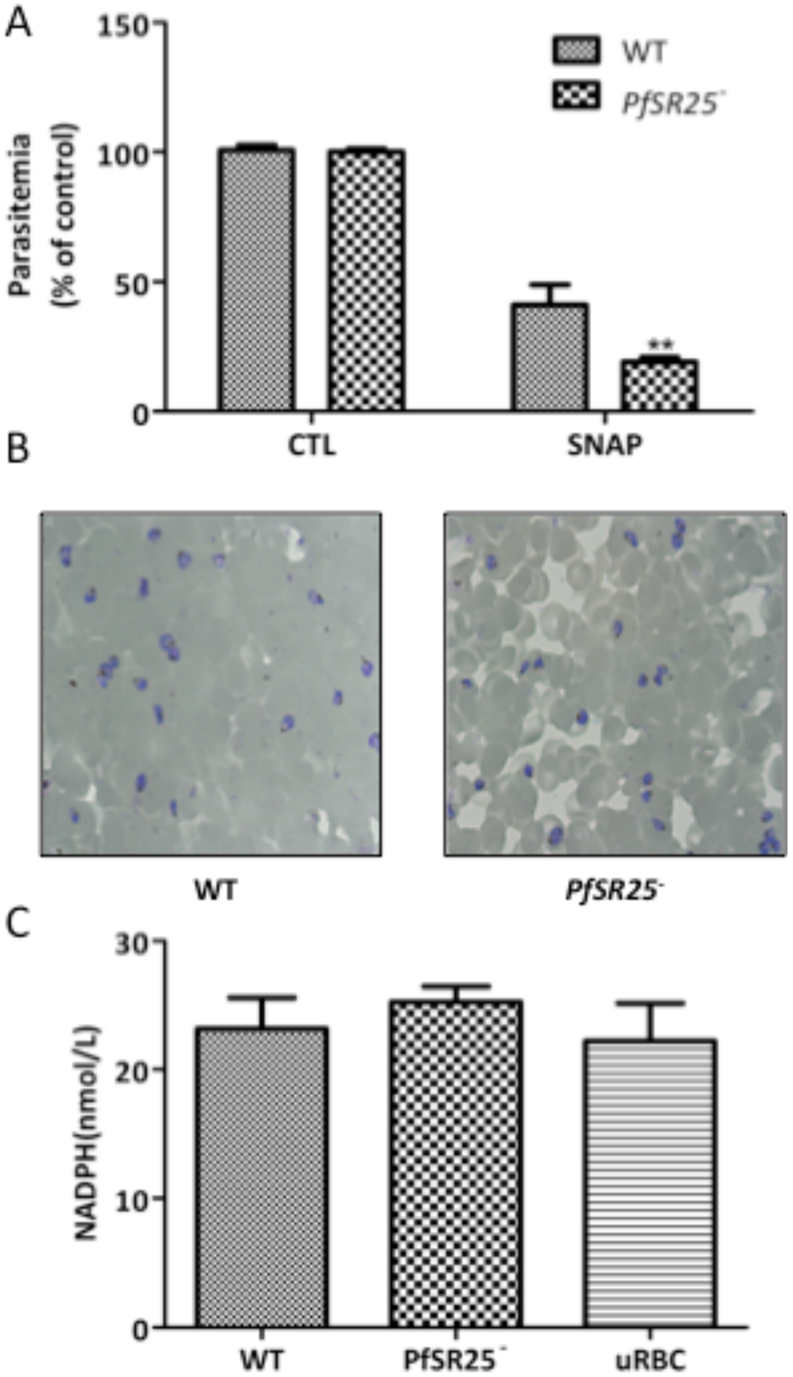

Fig. S4. Effect of SNAP and activity of G6PD on *P. falciparum*. (A) *P. falciparum* wt and *PfSR25*$^{-}$ were incubated under basal conditions (RPMI only) or with SNAP at concentration of 0.2 µM for 24 hours. Parasitemia was evaluated using DHE. **(B)** Giemsa stained image of the parasites used in the experiments. **(C)** 10^8 erythrocytes infected mature trophozoites were used to determine the cellular NADPH as described in methods. The data were expressed as percentages of those of the control conditions (100%), and represent the mean ± standard error of 3 different experiments performed in triplicate. $**p < 0.001$ compared to the control. uRBC, uninfected erythrocytes.

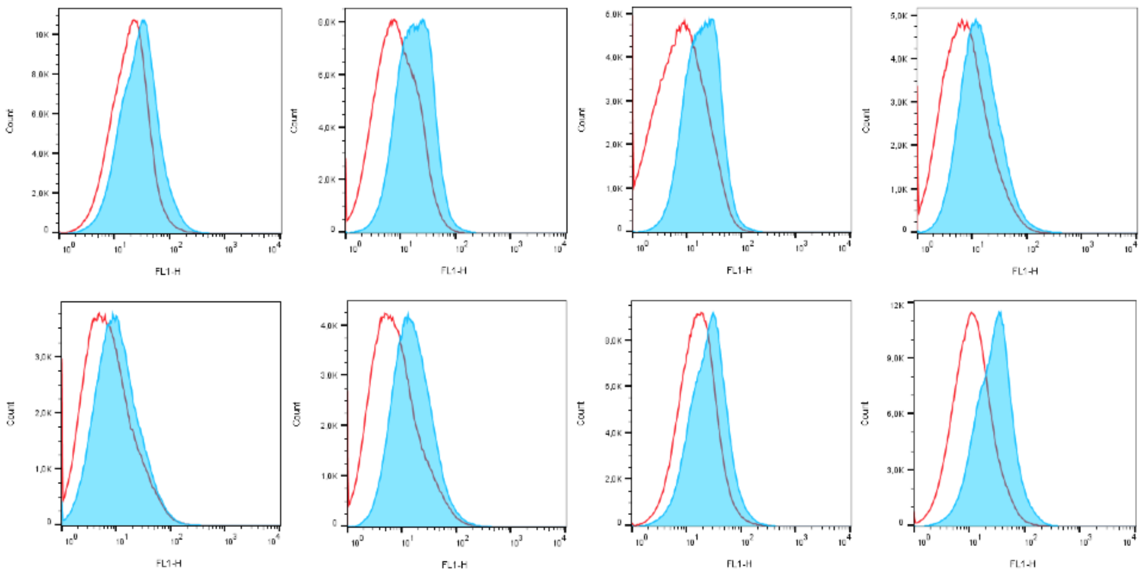

Fig. S5. Overlay diagram of shift in high K^+ (red) and high to low K^+ (cyan) in 3D7 parasite in presence of 2 mM Ca^{2+}

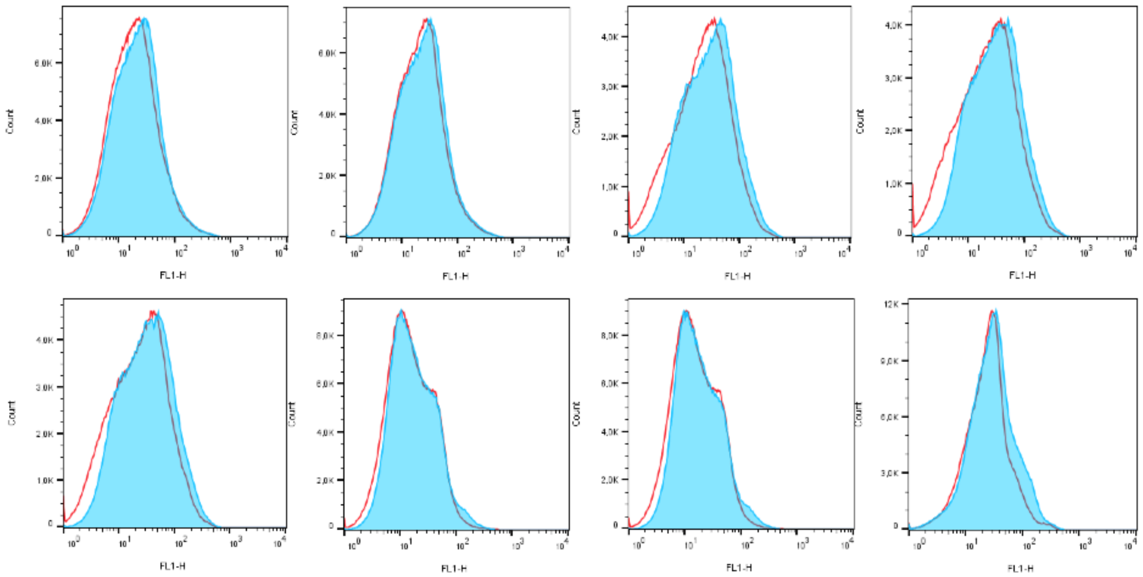

Fig. S6. Overlay diagram of shift in high K^+ (red) and high to low K^+ (cyan) in *PfSR25*$^-$ parasite buffer in presence of 2 mM Ca^{2+}

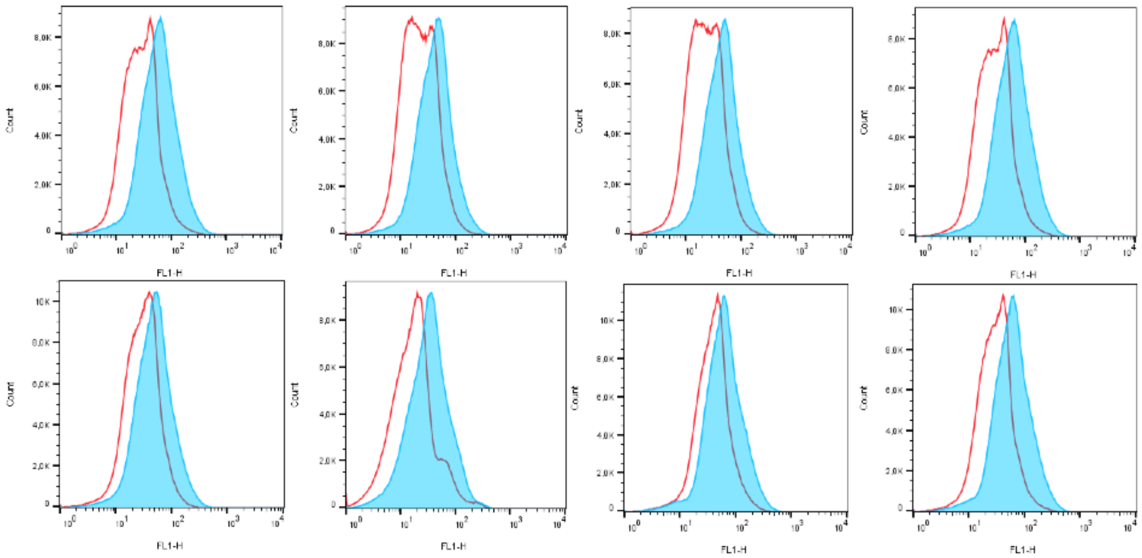

Fig. S7. Overlay diagram of shift in high K^+ (red) and high to low K^+ (cyan) in 3D7 parasite in Ca^{2+}-free buffer

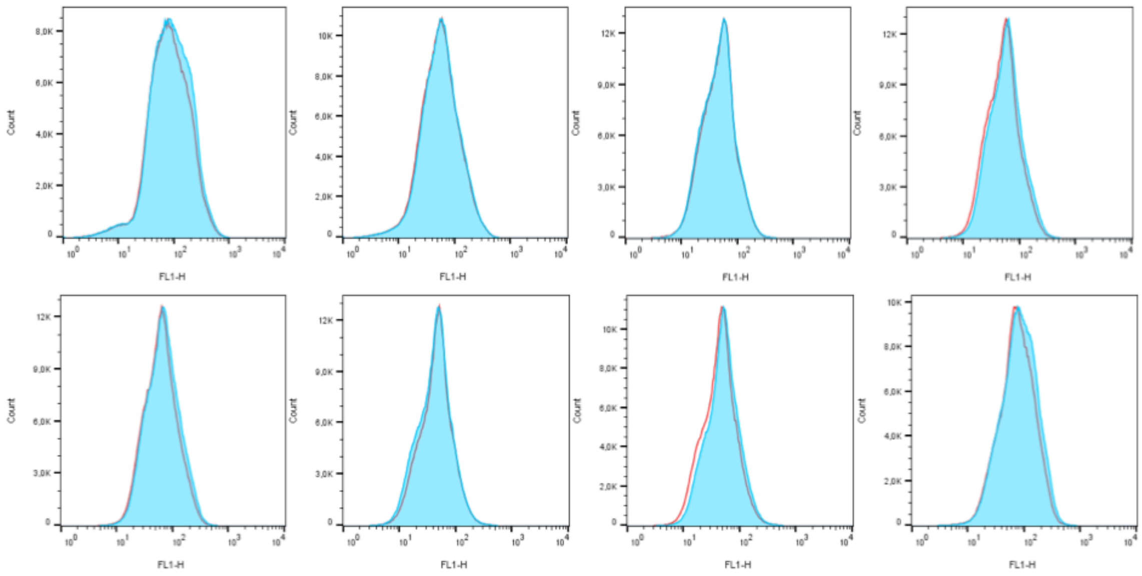

Fig. S8. Overlay diagram of shift in high K^+ (red) and high to low K^+ (cyan) in 3D7 parasite in presence of 10 μM CPA and 2 mM Ca^{2+}

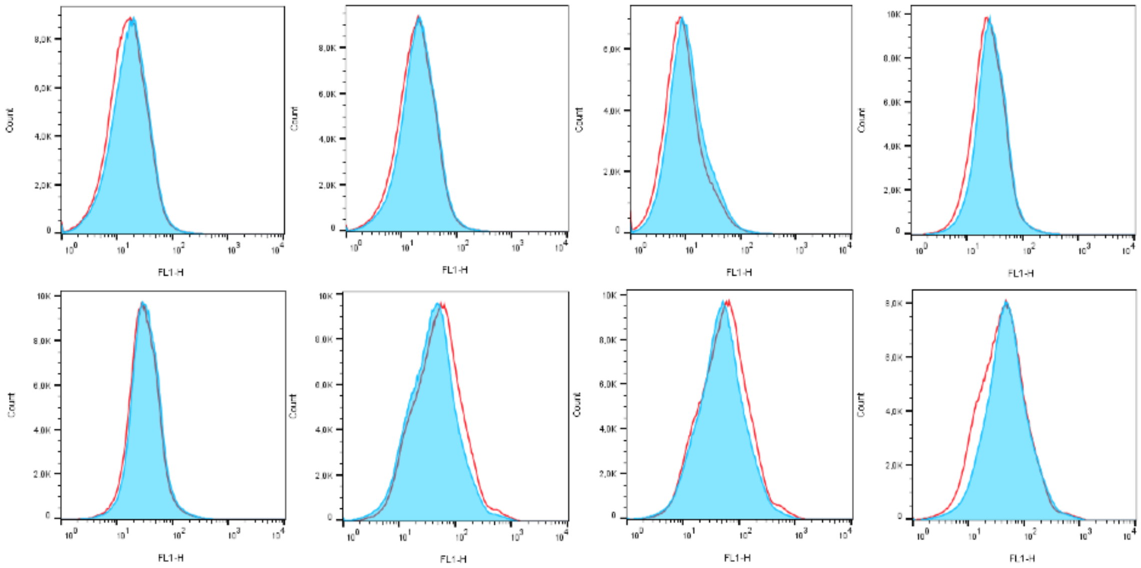

Fig. S9. Overlay diagram of shift in high high K^+ (red) and high to low K^+ (cyan) in 3D7 parasite pretreated with 1μM U73122, a PLC inhibitor in presence of 2 mM Ca^{2+}

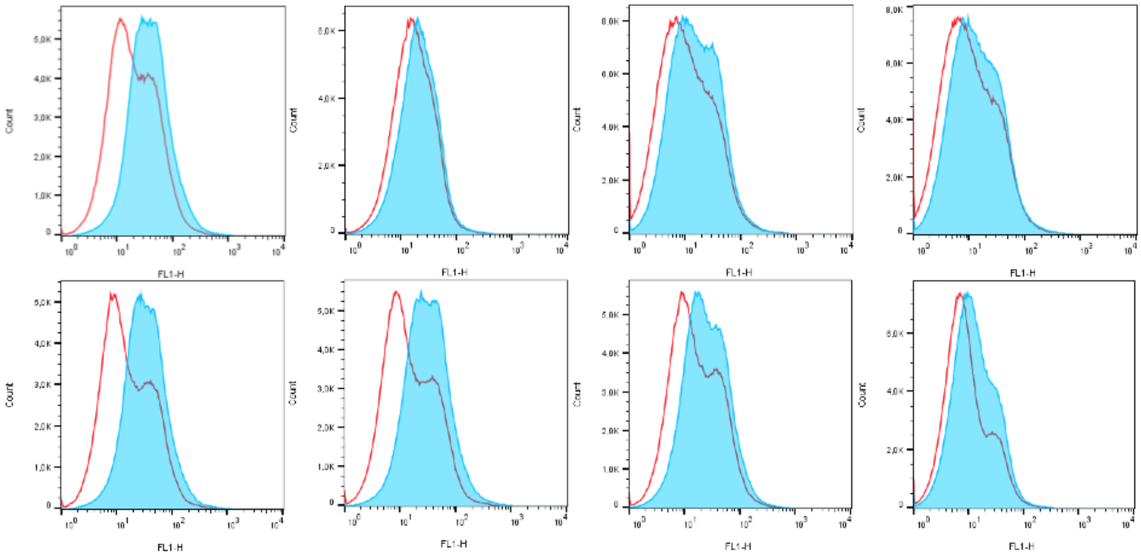

Fig. S10. Overlay diagram of shift in high K^+ (red) and high to low K^+ (cyan) in 3D7 parasite pretreated with 1μM U73343, inactive form of U73122 in presence of 2 mM Ca^{2+}

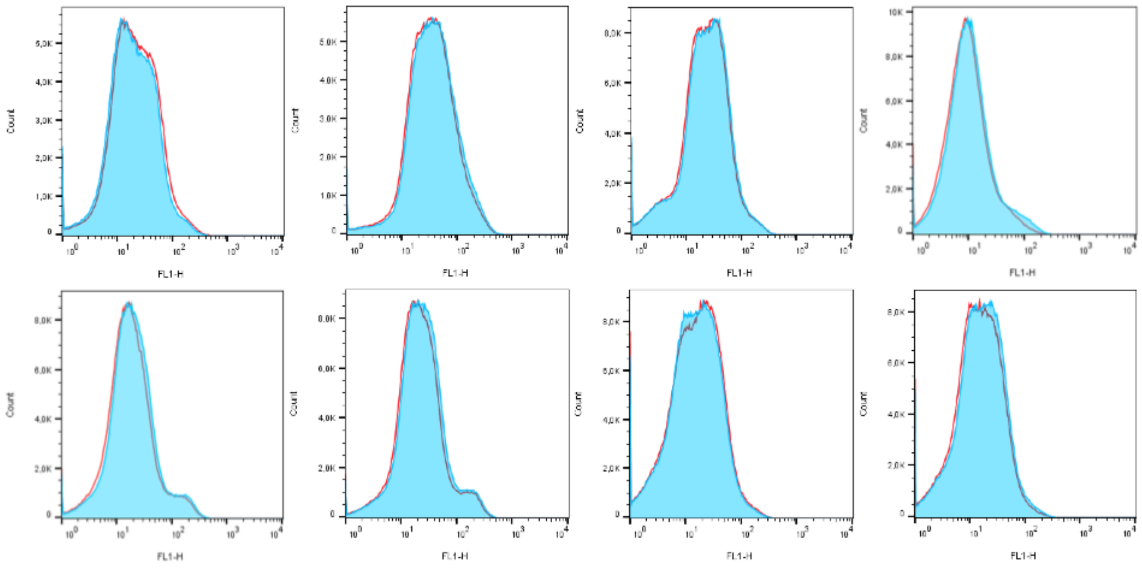

Fig. S11. Overlay diagram of shift in low K^+ (red) and low to high K^+ (cyan) in 3D7 parasite in Na-Gluconate buffer in presence of 2 mM Ca^{2+}

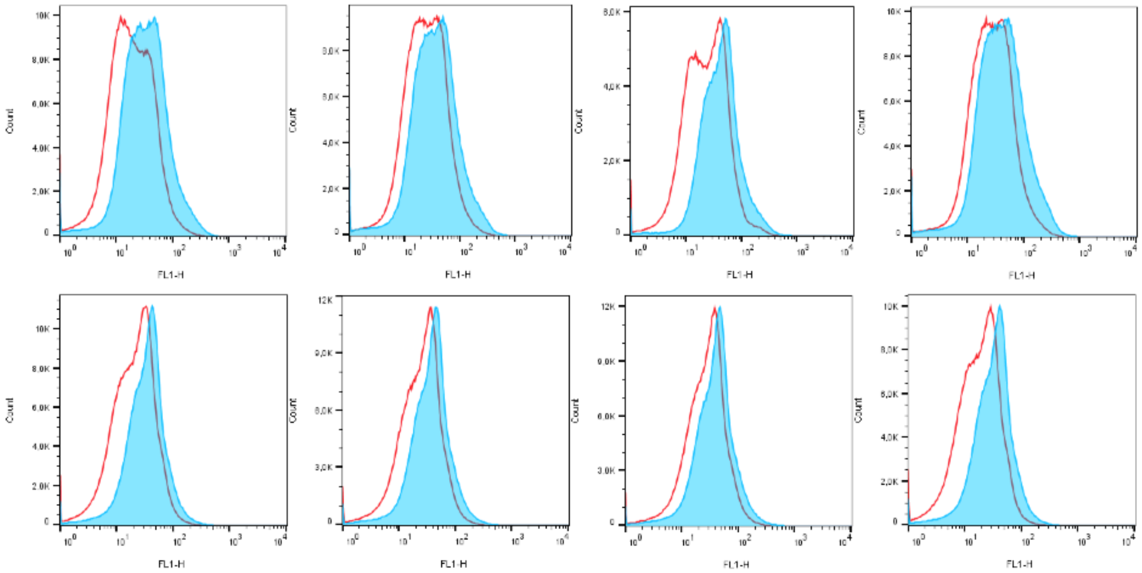

Fig. S12. Overlay diagram of shift in high K^+ (red) and high to low K^+ (cyan) in 3D7 parasite in K-Gluconate buffer in presence of 2 mM Ca^{2+}

Table 1. Cloning of the knocked out parasites after transfection.

**Intra venous injection (IV)**	**GFP+**	**Wild type**	**Parasitemia (day 9 post IV)**	**GFP/Wild type (%)**
**Sorting 1**				
Mouse 1	1600	20	3%	5/95
Mouse 2	1600	20	3%	5/95
**Sorting 2 ***				
Mouse 1	100	0.3	-	-
Mouse 2	500	1.5	-	-

*both mice didn't get infected after 1 month

After transfection only the 0.3% (final parasitemia 5%) of total parasites were GFP positive, meaning that they received the plasmid. Using sorting facilities it was possible to purify the parasites at the 80-90%. After a first round of sorting purification, 2000 parasites were injected in the vein of 2 mice, thus 20 wt and 1600 gfp were injected.

After 9 days, that means 9,5 cycles, when the parasitemia was 3%, only the 5% were gfp+ and 95% wt. That means that 20 wt parasites gave $3x10^8$ parasites, while 1600 GFP gave $1.5x10^7$ parasites suggesting that the multiplication rate of the knocked out parasites is the 35% of the wild type.

After a second sorting, it was injected 100 parasites (and 0.3 wt) and 500 parasites (and 1.5 wt) to reduce the wt contamination. Both mice never got infected. Thus, cloning and purification of mutant knocked out parasites was not possible.
